# The Recent Progress on Silver Nanoparticles: Synthesis and Electronic Applications

**DOI:** 10.3390/nano11092318

**Published:** 2021-09-06

**Authors:** Abderrhmane Bouafia, Salah Eddine Laouini, Abdelaal S. A. Ahmed, Alexander V. Soldatov, Hamed Algarni, Kwok Feng Chong, Gomaa A. M. Ali

**Affiliations:** 1 Department of Process Engineering and Petrochemistry, Faculty of Technology, University of Echahid Hamma Lakhdar El Oued, El-Oued 39000, Algeria; salah_laouini@yahoo.fr; 2 Chemistry Department, Faculty of Science, Al-Azhar University, Assiut 71524, Egypt; abdelaalsaiyd@gmail.com; 3 The Smart Materials Research Institute, Southern Federal University, Sladkova Str. 178/24, 344090 Rostov-on-Don, Russia; soldatov@sfedu.ru; 4 Research Centre for Advanced Materials Science (RCAMS), King Khalid University, P.O. Box 9004, Abha 61413, Saudi Arabia; halgarni@kku.edu.sa; 5 Department of Physics, Faculty of Sciences, King Khalid University, P.O. Box 9004, Abha 61413, Saudi Arabia; 6 Faculty of Industrial Sciences & Technology, University Malaysia Pahang, Gambang, Kuantan 26300, Malaysia; ckfeng@ump.edu.my

**Keywords:** silver nanoparticles, green synthesis, electronic applications, solar cells, gas sensors

## Abstract

Nanoscience enables researchers to develop new and cost-effective nanomaterials for energy, healthcare, and medical applications. Silver nanoparticles (Ag NPs) are currently increasingly synthesized for their superior physicochemical and electronic properties. Good knowledge of these characteristics allows the development of applications in all sensitive and essential fields in the service of humans and the environment. This review aims to summarize the Ag NPs synthesis methods, properties, applications, and future challenges. Generally, Ag NPs can be synthesized using physical, chemical, and biological routes. Due to the great and increasing demand for metal and metal oxide nanoparticles, researchers have invented a new, environmentally friendly, inexpensive synthetic method that replaces other methods with many defects. Studies of Ag NPs have increased after clear and substantial support from governments to develop nanotechnology. Ag NPs are the most widely due to their various potent properties. Thus, this comprehensive review discusses the different synthesis procedures and electronic applications of Ag NPs.

## 1. Introduction

Two thousand years ago, humans knew the medicinal properties of silver [1,2,3]. Silver particles have been used as antibacterial agents since the 19th century, and now their uses have diversified to include many new physical, chemical, and biological ones [4]. Ag NPs are considered to be one of the most widespread NPs, with about 500 tons of annual global production [5]. In the latest research, Ag NPs have been incorporated into industrial and surgical device coatings, dental coatings [5], and automotive smoke filters and textiles due to their effective properties against microbes [6]. The mechanism on this topic is currently under discussion by researchers [7].

In this review, the historical background of nanomaterials and the various synthesis techniques will be discussed. After that, a detailed study of the properties, synthesis, properties, and the various electronic applications of Ag NPs will be presented. This comprehensive review provides an insight into the different methods used in the synthesis and an overview of the applications of Ag NPs in the electronic field.

## 2. Metal Nanoparticles Synthesis

As a historical background for metal nanoparticles (MNPs), it was reported that many exploited the strengthening of ceramic matrices, including natural asbestos nanofibers, more than 4500 years ago [8]. Lead-based chemistry was pioneered in ancient Egypt for cosmetic preparation over 4000 years ago. Here, we look at a hair dye recipe using lead salts described in the text since Greco-Roman times. We report direct evidence for the shape and distribution of PbS nanocrystals that form in the hair during darkening [9]. Likewise, “Egyptian blue” was the first synthetic pigment prepared and used by the Egyptians using a mixture of sintered nanometer-scale glass and quartz around the 3rd century BC [10]. Egyptian Blue represents a complex mixture of CaCuSi_4_O_10_ and SiO_2_ (both glass and quartz). In the ancient geographic regions of the Roman Empire, including Egypt, Mesopotamia, and Greece, Egyptian blue for decorative purposes has been observed during archaeological excavations.

**Figure 1:**
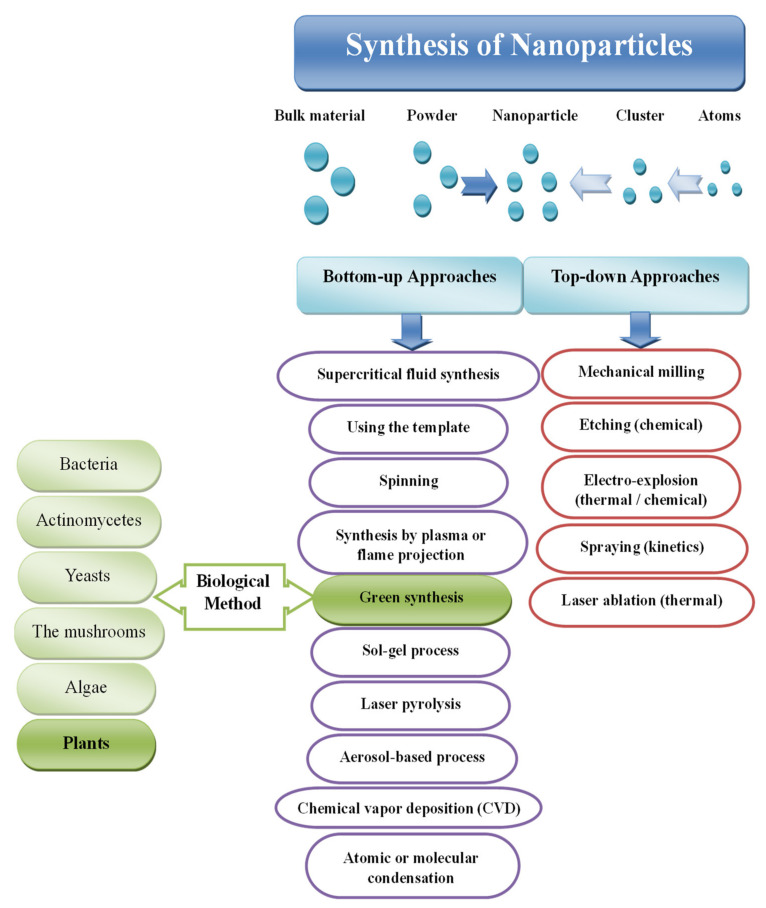
Techniques for synthesis metal NPs, modified with permission from Ref. [17], MDPI, 2019.

Natural or synthetic ways can synthesize NPs by two basic approaches, including various sub-preparation methods (Figure 1). The first approach is called the "top to bottom" method, including breaking down bulk solid materials into smaller pieces by applying external energy from physical, chemical, and thermal techniques [11]. The second approach, called "from the bottom up, "brings together and combines atoms or molecules of gases or liquids. The top-down approach is expensive. However, it is impossible to obtain perfect surfaces and edges due to the cavities and roughness in NPs, while a bottom-up approach can get excellent results of nanoparticle synthesis. In addition, with the bottom-up approach, no waste is formed to be eliminated, and the smaller NPs can be obtained with better control of the sizes [12,13,14,15,16].

However, adapting the production of large quantities of powders on an industrial scale is not simple. A significant advantage of treatment in solution is the possibility of generating encapsulated NPs using surfactants as a protective shell, which makes it possible to obtain very homogeneous and well-dispersed NPs [18]. Surfactants are amphiphilic compounds with a polar head group and one or more hydrophobic hydrocarbon chains. Alkyl thiols, long-chain amines, carboxylic and phosphonic acids, phosphine and phosphine oxides, phosphates, phosphonates various coordination solvents (e.g., ethers, THF, DMF) or not (e.g., alkanes, alkenes) have been widely investigated as a colloidal synthesis.

### 2.1. Sol-Gel Method

Sol-gel technique is widely used to produce metal oxide nanostructured materials in technical and technological applications [19,20,21,22]. This is mainly assigned to the controlled shape and size of the obtained nanomaterials. Since the synthesis of silica gel by Ebelman in 1846, this method has been developed in various applications with excellent optical, magnetic, electrical, thermal, and mechanical properties [23]. The synthesis of solid materials usually involves wet chemistry reactions and sol-gel chemistry based on the transformation of molecular precursors into a network of oxides by hydrolysis and condensation [21,24,25]. Morphology is crucial in developing material properties by enhancing the surface/volume ratio. Controlling material particles’ shape, size, and packaging structure is vital in constructing next-generation therapeutic devices and materials [23,26]. A sol-gel procedure is an exceptional tool for deploying a controlled architecture in materials chemistry to fabricate metal oxide nanostructures (NSOMs). Metal oxides prepared in sol-gel solution possess excellent optical and electrical properties. Examining important NSOMs derived from the sol-gel method helps us understand the factors that control particle shape and size. In general, solvents, additives, aging time, and heat post-treatment are crucial factors determining the shape and size of the building blocks of synthesized materials [27].

**Figure 2:**
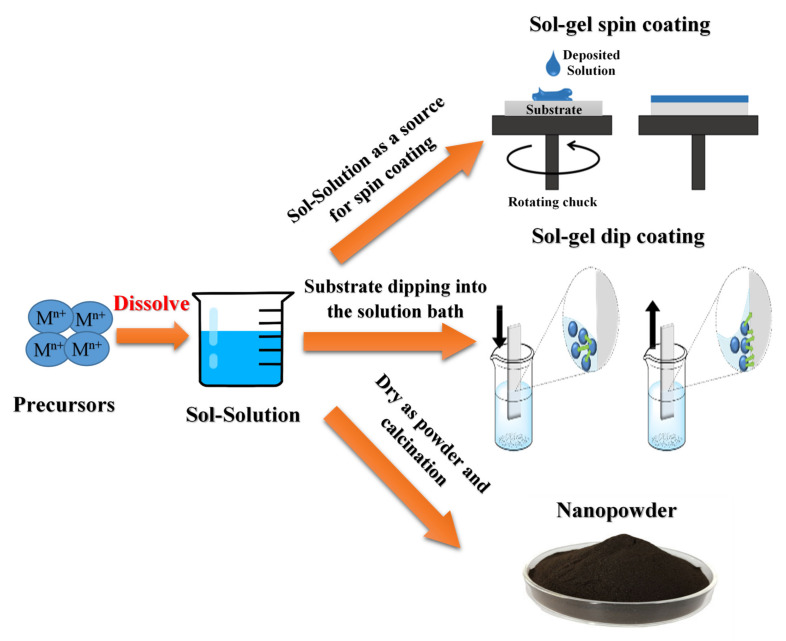
The representative diagram of the sol-gel technique, modified with permission from Ref. [29], IntechOpen, 2017.

Figure 2 shows a representative diagram of the sol-gel method. The sol is obtained by hydrolysis or polymerization reactions by adding appropriate reagents to the precursor solution. The obtained sol can be deposited on a surface of the substrate from a thin film by spin-coating or dip-coating techniques. Typically, the gelation process condenses the sol or adds polymers to convert the sol into a gel. The obtained gel can be used to form various materials such as NPS, xerogel, glass, or ceramics, depending on the subsequent processing steps involved. Both NPs and xerogels can be obtained by simple evaporation of the solvent. The obtained xerogel can be formed into ceramic form by heat treatment, and the glassy nature can be induced by melting techniques. Thus, the sol-gel process can obtain different materials with a controlled phase, shapes, and sizes [28].

### 2.2. Hydrothermal Method

**Figure 3:**
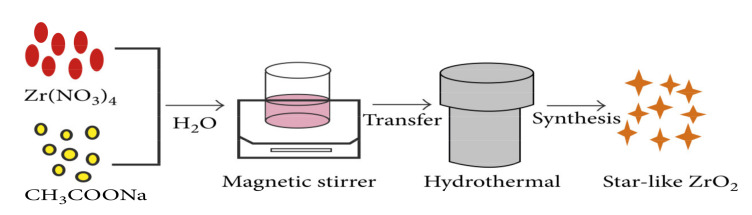
The procedure for ZrO_2_ preparation via the hydrothermal method, copied with permission from Ref. [32], Hindawi, 2018.

Hydrothermal synthesis is an approach based on the reaction in solution, as shown in Figure 3. Generally, the hydrothermal process can be defined as the preparing materials in a temperature ranging from room temperature to high-temperature solutions [30,31]. Depending on the vapor pressure of the main composition in the reaction, the applied pressure should be controlled to investigate the influence on the morphology of the prepared materials. Hydrothermal synthesis can generate nanomaterials that are not stable at high temperatures; however, the hydrothermal method can produce high vapor pressure nanomaterials with minimal material loss. In this method, the compositions of the synthesized nanomaterials can be controlled by liquid phase or multiphase chemical reactions.

### 2.3. Green Synthesis Method

**Figure 4:**
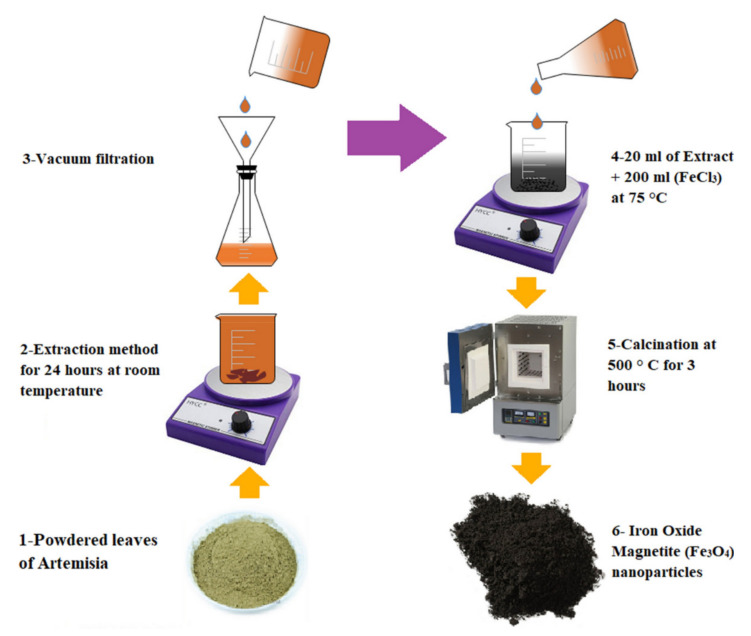
Green synthesis for Fe_3_O_4_ nanoparticles, copied with permission from Ref. [36], Springer-Nature, 2020.

Green synthesis approaches have attracted great attention in developing nanomaterial preparations to overcome the limitations of the techniques mentioned above [33,34,35]. The procedure for this technique is described in Figure 4. The green synthesis of nanomaterials, produced by regulation, control, cleaning, and remediation, will directly help improve their environmental friendliness. Some basic principles of “green synthesis” can therefore be explained by several components such as waste minimization, pollution reduction, and the use of safer (or non-toxic) solvents as well as renewable raw materials.

Green syntheses are necessary to avoid unwanted or harmful by-products by establishing reliable, sustainable, and environmentally friendly synthesis procedures. The use of ideal solvent and natural resource systems (such as organ systems) is essential to achieve this goal. The green synthesis of metal nanoparticles has been adopted to accommodate various biological materials (e.g., bacteria, fungi, algae, and plant extracts). Among the environmentally friendly methods available for synthesizing metal oxide nanoparticles, the use of plant extracts is a straightforward process for producing NPs on a large scale compared to the synthesis mediated by bacteria and/or bacteria and mushrooms.

## 3. Silver Nanoparticles

According to the Royal Society of Chemistry, the first evidence of silver mining dates to 3000 BC in Turkey and Greece. Even the ancients knew how to polish silver, heat silver ore, and blow air on it. Silver does not react with air, but base metals such as lead and copper oxidize and separate from precious metals. Silver, like gold, is formed from the explosion of a star called a supernova. A 2012 study published in Astronomy and Astrophysics found that exploding small stars produce silver and large stars. When Europeans arrived in the New World in 1492, silver was abundant on Earth. Spanish invaders have enthusiastically drawn this wealth by discovering that South America has silver- and ore-rich veins. According to the Silver Institute, 85% of the silver produced worldwide came from Bolivia, Peru, and Mexico between 1500 and 1800.

**Figure 5:**
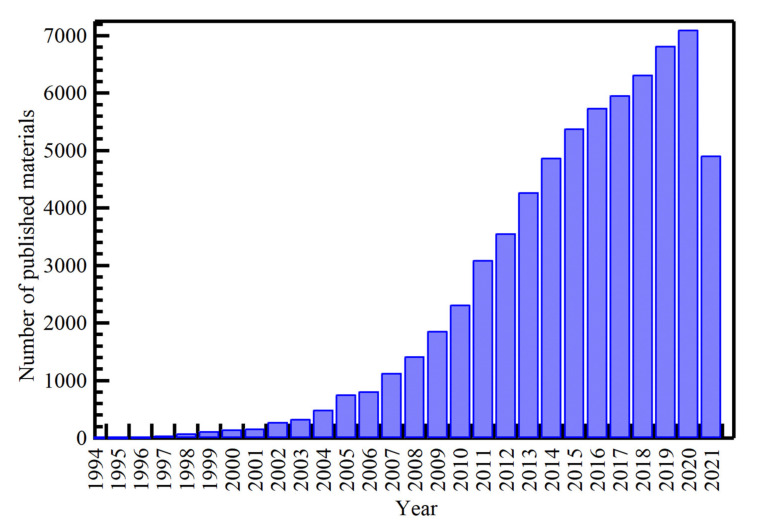
Publications distribution with time on Ag NPs.

Among the various types of NPs, Ag NPs have been widely developed to be utilized in various applications due to their outstanding properties. Typical applications of Ag NPs include clothing and textiles, medical devices, food storage, cosmetics, sunscreens, laundry detergents [37], bandages [38], and sensors [39]. Some studies have found that Ag NPs have cytotoxicity that can induce ROS formation in cells [40]. Therefore, many products such as detergents, toiletries, etc. In addition, their synthetic or in-use personal care, whether industrial or household, produces a release of NPs, which ultimately ends up in the sewer. This untreated wastewater affects aquatic ecosystems and thus microorganisms. Recently, Ag NPs have great concerns regarding aquatic toxicology due to the difficulty of tracking these particles in the environment and accessing their effects on living organisms [41]. The fate of NPs in the aquatic environment and their interactions, the interactions between NPs with biological and abiotic components, and their potential for damage are not well understood, and these uncertainties raise concerns about related risks. These molecules impose on humans on health and the environment [42]. Based on the Scopus database [43], the publications on Ag NPs increase with time, where it started in 1990 (two reports) and reached 7105 reports in 2020, as shown in Figure 5.

### 3.1. History of Silver Nanoparticles

Colloidal Ag NPs are molecules with an average diameter of 20–40 nm and comprise 80% silver atoms and 20% silver ions. They are the best-selling nanoparticles ahead of carbon nanotubes and titanium nanoparticles and are released into the environment. The demand for Ag NPs has increased due to their applicability in multiple fields. Over the years, various synthesis techniques have been developed, and procedures have been improved to prepare small and uniform Ag NPs. Ag NPs were prepared by a chemical reduction technique, where the silver ions are reduced by sodium citrate [44].

Further investigations into the role of citrate revealed that besides acting as a reducing agent, the citrate anion also has a proven effect on Ag NPs. At this stage, the LaMer nucleation and growth mechanism was used to characterize the nucleation and growth of the nanoparticles. However, studies revealed that LaMer’s intent and growth [45]. Mechanism not related to their feedback. Therefore, Fan Hing et al. examined the mechanism of nucleation and growth that applied to its results. They prepared Ag NPs using a chemical reduction technique in which silver perchlorate ions are reduced by sodium borohydride. It was determined in their study that increasing the concentration of sodium borohydride would lead to the instability of Ag NPs.

In the study conducted by Zielińska et al. [46], the stability of Ag NPs was the main focus. Ag NPs were prepared using the chemical reduction method. Zielińska et al. show that the type of precursor affects particle stability. According to their research, stable and spherical Ag NPs were synthesized using silver nitrate as a precursor in the presence of NaBH_4_ as a reducing agent and stabilizer. These results were compared with Ag NPs prepared using silver nitrate or silver acetate as a precursor. In this case, the Ag NPs were unstable and precipitated shortly after synthesis. Alternatively, their study presented that it is also possible to synthesize stable and uniform spherical Ag NPs via chemical reduction technique using AgNO_3_ as a precursor, NaBH_4_ as a reducing agent, and polymer as a stabilizing agent [47].

In a study conducted by Hsu and Wu [48], crystalline Ag NPs with a particle size in the range of 3–15 nm were prepared by reducing silver ions by formaldehyde using three stabilizers. Reduction by formaldehyde is relatively slow and requires a catalyst or reaction catalyst to speed up the reaction process. Moreover, the synthesis at high pH favors a decrease in efficiency in the presence of poly (N-vinyl-2-pyrrolidone) (PVP), resulting in spherical Ag NPs with an average particle size of 15 nm. Thiosalicylic acid as a stabilizer will produce spherical Ag NPs with a size of 8 nm. Finally, triethylamine (TEA) has the smallest spherical 3 nm Ag NPs. It was observed that without the addition of a stabilizer, the particles would agglomerate, which was inhibited by Ag-N bonding by TEA. Adjusting the stabilizer concentration is critical, as increasing the TEA concentration increased particle agglomeration [49]. Sánchez et al. produced Ag NPs in the application of 20 V to silver electrodes. Another observation is that complete cathode coverage limits the formation of NPs and thus favors the particle deposition process. To avoid cathode precipitation and reduction in nanoparticle production [50], two metallic silver plates as an electrode pair were used to synthesize colloidal Ag NPs. Laser wavelength on particle morphology was investigated. They showed that the deposition of metallic Ag NPs onto a silica substrate by laser ablation under vacuum resulted in reduced particle size as the laser wavelength decreased [51].

### 3.2. Synthesis of Silver Nanoparticles

#### 3.2.1. Top-Down and Bottom-Up Approaches

**Figure 6:**
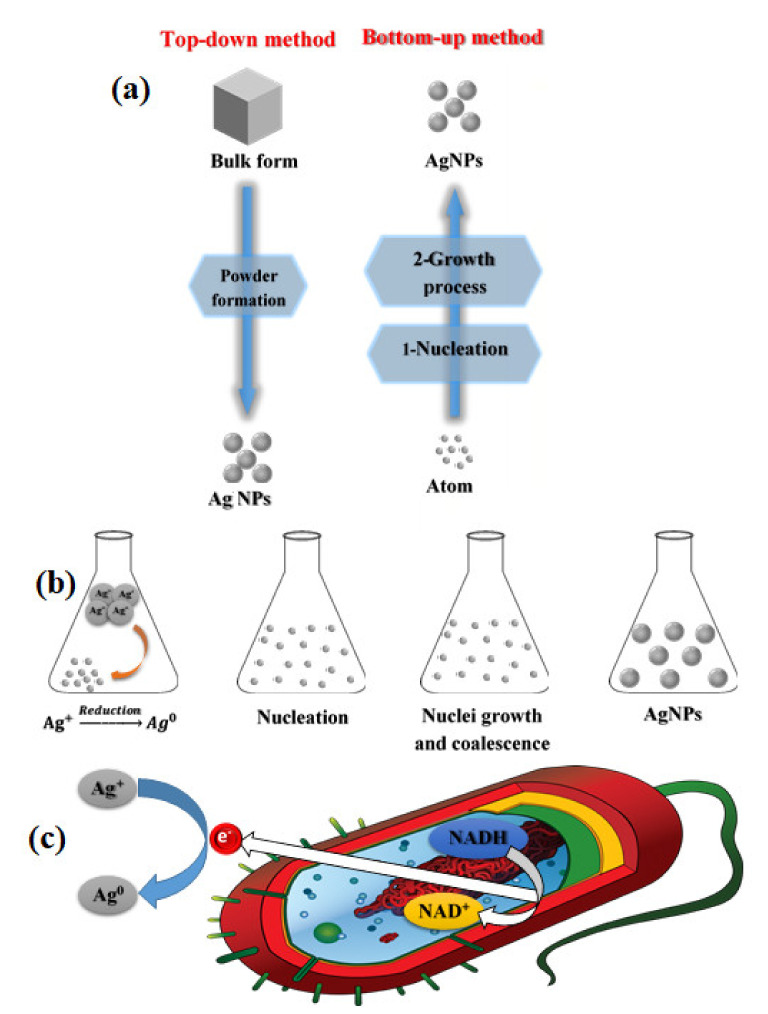
Synthesis routes of Ag NPs by (**a**) Top-down and bottom-up methods, (**b**) chemical methods, and (**c**) green methods, modified with permission from Ref. [55], MDPI, 2019. The electron transfer initiates the bioreduction through nicotinamide adenine dinucleotide (NADH)-dependent reductase as an electron carrier to form NAD^+^. The resulting electrons are obtained by Ag^+^ ions, which are reduced to elemental Ag NPs.

As referenced, various kinds of Ag NPs have been utilized in various applications [52]. Specifically, Ag NPs of differing sizes and shapes have been used in a broad scope of uses and clinical gear, such as electronic gadgets, coatings, cleansers, cleansers, swathes, etc. [53]. Explicit physical and optical properties of Ag NPs are subsequently essential factors in advancing their use in these applications. In such a manner, the accompanying subtleties of the materials are imperative to consider in their combination: surface property, size dissemination, clear morphology, molecule composition, dissolution rate (i.e., reactivity in arrangement and effectiveness of particle delivery), and kinds of diminishing and capping specialists utilized. The blend techniques for metal NPs are partitioned into top-down and bottom-up approaches, as demonstrated in Figure 6.

The big picture perspective disincorporates mass materials to produce the required nanostructures, while the base-up strategy gathers single particles and molecules into bigger nanostructures to create nanosized materials [54]. The following areas examine different combination strategies in detail, from the union of spherical Ag NPs to shape-controlled Ag colloids, just as size-controlled Ag NPs are synthesized. The segments also expect to present different courses of union and their instruments, elucidating how shape-and size-controlled amalgamation of Ag NPs can be accomplished through the fitting choice of energy source, antecedent synthetic substances, diminishing and covering specialist, just as well fixation and the molar ratio of synthetic compounds.

#### 3.2.2. Physical Methods

The physical technique usually utilized to prepare Ag NPs is the evaporation-condensation method. It is commonly performed using a tube furnace at atmospheric pressure, synthesizing various sizes [56]. Several attempts have been made. For instance, Tsuji et al. [57] proposed a new method for synthesizing Ag NPs by a laser ablation technique with focused and unfocused laser beam irradiation carried out at 12 and 900 mJ cm^−2^ intensities, respectively. The radiation wavelengths used were 355, 532, and 1064 nm. This study revealed that the surface plasmon wavelength of Ag NPs irradiated using 355, 532, and 1064 nm is ~400 nm for focused and unfocused beams.

The physical synthesis of Ag NPs incorporates the evaporation-condensation way and the laser ablation method (Figure 6) [58]. Both approaches can synthesize enormous quantities of Ag NPs with high immaculateness without using chemicals that discharge poisonous substances and endanger human health and climate. Notwithstanding, agglomeration is regularly an incredible test due to the absence of capping agents. Moreover, both approaches devour more noteworthy power and generally need a longer production and complex hardware, increasing the total production cost.

Evaporation–condensation and laser ablation are the principal physical attitudes. The shortfall of solvent contamination in the readied thin films and the consistency of NPs conveyance are the upsides of physical synthesis methods compared to the chemical processes. The biological synthesis of Ag NPs utilizing a tube furnace at an atmospheric pressing factor has a few drawbacks. The tube furnace consumes a considerable space, burns through a great measure of energy while raising the ecological temperature around the source material, and requires a ton of time to accomplish warm steadiness. Additionally, a commonplace tube furnace needs power utilization (a few kilowatts) and several minutes of preheating to arrive at a stable operating temperature [59]. It was revealed that Ag NPs could be synthesized using a small ceramic heater with a neighborhood heating zone [60]. The little ceramic heater was used to evaporate source materials. The evaporated fume can cool quickly because the temperature angle near the heater surface is exceptionally steep in examining a tube furnace.

Furthermore, Ag NPs sizes obtained from the laser ablation with nanosecond and femtosecond laser beats were analyzed. In the arrangement, the wavelength of the laser utilized was 800 nm. Additionally, a Ti: sapphire laser system created femtosecond laser beats used in the examination [61]. It was tracked that Ag NPs sizes retrieved from the nanosecond laser beats were more modest than those from femtosecond laser beats were. The average widths of the obtained Ag NPs from nanosecond and femtosecond laser beat schemes were 27 and 41 nm. For technique enhancement, synthesizing Ag NPs by condensation utilizing a small ceramic heater with a nearby warming area of 500 m^2^ was projected [60]. Unadulterated spherical Ag NPs with shifting measurements from 6.2 to 21.5 nm were effectively created. The examination inferred that the geometric mean breadth, the geometric standard deviation, and the absolute number centralization of Ag NPs increased with the surface temperature of the heater. Their approach could dependably synthesize stable Ag NPs, since the heater surface temperature did not fluctuate with time [60]. Various Ag NPs prepared by physical methods are listed in Table 1.

#### 3.2.3. Chemical Methods

**Figure 7:**
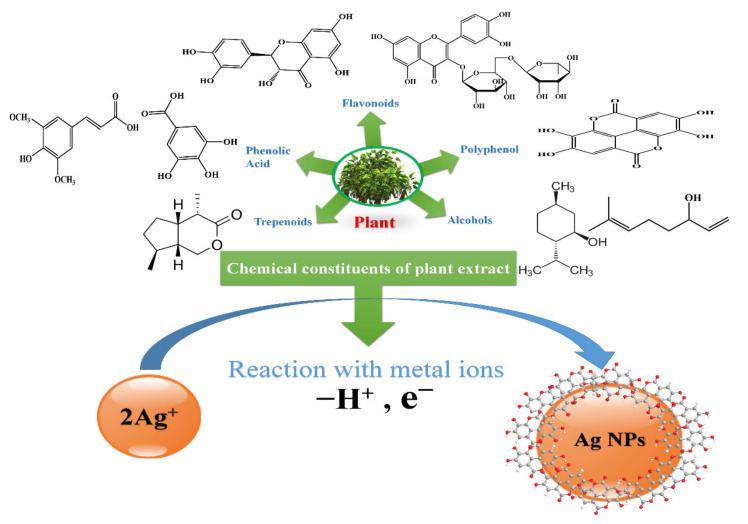
Ag NPs synthesis mechanism from plant extract, modified with permission from Ref. [73], Royal Society of Chemistry, 2019.

The famous method for Ag NPs synthesis is a reduction by natural and inorganic reducing agents (Figure 7). By and large, unique reducing agents, for example, sodium citrate, ascorbate, sodium borohydride, essential hydrogen, polyol measure, Tollens reagent, N, N-dimethylformamide (DMF), and poly (ethylene glycol)-block copolymers are utilized for the reduction of silver particles (Ag^+^) in aqueous or non-aqueous arrangements. These reducing agents decrease Ag^+^ and lead to metallic silver (Ag^0^), trailed by agglomeration into oligomeric clusters. These clusters ultimately arrange the metallic colloidal silver particles [71]. It is critical to utilize defensive agents to stabilize dispersive NPs during metal nanoparticle planning and ensure that the NPs consumed or tied onto nanoparticle surfaces stay away from their agglomeration [72].

Surfactants involving collaborations with molecule surfaces can stabilize molecule growth and shield particles from sedimentation, agglomeration, or losing their surface properties. Stabilizing dispersive NPs during Ag NPs synthesis is critical. The most well-known methodology utilizes stabilizing agents that can be absorbed outside Ag NPs, evading their agglomeration [74]. To stabilize and to dodge agglomeration and oxidation of NPs, capping agents can be utilized, for example chitosan, oleylamine gluconic acid, cellulose or polymers such as poly N-vinyl-2-pyrrolidone (PVP), polyethylene glycol (PEG), polymethacrylic acid (PMAA) and polymethylmethacrylate (PMMA) [75]. Adjustment using capping agents can be accomplished either through electrostatic or steric shock. For example, electrostatic adjustment is typically achieved through anionic species such as citrate, halides, carboxylates, or polyoxoanions that adsorb or cooperate with Ag NPs to bestow a negative charge on the outside of Ag NPs. Therefore, the surface charge of Ag NPs can be constrained by covering the particles with citrate particles to give a solid negative charge. Contrasted with utilizing citrate particles, fanned polyethyleneimine makes an amine-functionalized surface with a profoundly specific charge. Other capping agents likewise give extra usefulness. Polyethylene glycol (PEG)-coated NPs show excellent stability in exceptionally salty solutions, while lipoic acid-coated particles with carboxyl gatherings can be utilized for bioconjugation.

#### 3.2.4. Green Methods

Green synthesis methodologies dependent on natural reducing agents rely on different reaction parameters such as solvent, temperature, pressing, and pH conditions (acidic, fundamental, or impartial). For the union of metal oxide nanoparticles, plant biodiversity has been extensively viewed as the accessibility of successful phytochemicals in different plant separates, particularly in leaves such as ketones and aldehydes flavones, amides, terpenoids, carboxylic acids, phenols, and ascorbic acids [36,76]. These parts are fit for decreasing metal salts into MNPs [77]. The fundamental focus of such nanomaterials has been researched for biomedical diagnostics, antimicrobials, catalysis, atomic detecting, optical imaging, and marking of natural frameworks.

Essential elements for the green synthesis of Ag NPs are silver salts and bioreducing agents [78]. In general, bioreducing agents or various components present in cells act as stabilizers or capping agents, reducing these agents’ need for external inclusion [79]. Traditional strategies for producing NPs are costly, harmful, and not environmentally friendly. Therefore, to overcome these problems, experts are adopting a green method for synthesizing NPs. Natural resources and their constituents were used in the synthesis of NPs. Generally, plants and their extracts, bacteria, fungi, and biopolymers can prepare Ag NPs via the green technique, as shown in Table 2. The green composition of plants, plant extracts, bacteria, fungi, and overall biopolymers is discussed in Section 3.2.5 of this review.

#### 3.2.5. Green Synthesis by Plant Extract

The plant-based synthesis of Ag NPs is largely embraced more in contrast to techniques that utilize microorganisms, since it tends to be developed effectively, is less biocompromising, and excludes the progression of cell culture growth [80]. Leaves, natural products, roots, seeds, and stems contain biomolecules such as compounds, alkaloids, polysaccharides, tannins, terpenoids, phenols, and nutrients of extraordinary restorative worth despite their complex structures, are helpful for the environment [81,82]. Plant extract replaces all harmful synthetic compounds such as trisodium citrate and sodium borohydride. The concentrate from plants helps well in the amalgamation of NPs because of the arrangement of Ag NPs settled by the flavonoid and terpenoid parts present in leaf stock, while the decrease of silver particles is assigned by the polyol water-solvent heterocyclic segments of leaf broth [83]. The concentrate of plant *Salvia spinosa* under in vitro conditions was utilized interestingly to incorporate Ag NPs [84]. The preparation of Ag NPs by Alfalfa sprouts was firstly introduced [85].

These days, the creation of NPs centers around green synthesis from the extract of various plant parts [86]. The multipurpose agents of reduction and adjustment of plant extraction for biological synthesis of NPs are used to execute green science [87]. Extraction of nontoxic plants for synthesis nanoparticles offer characteristic capping agents. Besides, as far as the cost for nanoparticle synthesis, plant extraction improves the cost viability over the disconnection of microorganisms for the achievability of nanoparticle synthesis [88]. As of late, there is a growing interest in synthesizing MNPs by ‘green’ techniques.

For this reason, extracts of various plants have been used for Ag NPs preparation. One of the primary approaches to utilizing plants as a source for the synthesis of MNPs was with Alfalfa sprouts [85], which was the main report on the arrangement of Ag NPs using a living plant system. Hay roots can engross Ag from agar medium and move them into the plant’s shoots in a similar oxidation state. These Ag molecules orchestrated themselves in the shoots to shape NPs by joining themselves and framing bigger courses of action.

In contrast with bacteria and fungi, green synthesis utilizing plants has all the earmarks of being quicker, and the principal examinations exhibit that synthesis methodology can produce Ag NPs quite quickly. For example, Shankar et al. showed that utilizing *Geranium* leaf takes around nine hours, arriving at 90% response contrasted with the 24 to 124 h vital for other responses announced before [89]. Therefore, the utilization of plant extracts in green synthesis has prodded various examinations and studies up till now. It was shown that the creation of MNPs utilizing plant extracts could be finished in the metal salt arrangement inside the space of minutes at room temperature, contingent upon the idea of the plant extract. After the decision of the plant extract, the primary influencing boundaries are the grouping of the extract, the metal salt, temperature, pH, and contact time [90]. The mechanism of preparing Ag NPs from plant extract is shown in Figure 7. In addition, Table 3 lists the synthesis of Ag NPs using plant extracts with different sizes, shapes, characterization, and applications.

#### 3.2.6. Green Synthesis Using Bacteria

As of late, the capability of biosynthesis of Ag NPs using bacteria has been acknowledged [99]. For example, *Pseudomonas stutzeri* AG259, disconnected from silver mine, was utilized to deliver Ag NPs inside the cells [100]. In addition, a few bacterial strains (Gram-negative just as Gram-positive), specifically *A. calcoaceticus*, *B. amyloliquefaciens*, *B. flexus*, *B. megaterium,* and *S. aureus* have been utilized for both extra-and intracellular biosynthesis of Ag NPs [101]. These Ag NPs are spherical, disk, cuboidal, hexagonal, and triangular fits. Saifuddin et al. [102] have exhibited an extracellular biosynthesis of Ag NPs (∼5–50 nm) using a mix of culture supernatant of B. subtilis and microwave light in water. Shahverdi et al. [103] have detailed quick biosynthesis of Ag NPs (inside five min) using the way of life supernatants of *K. pneumonia*, *E. coli,* and *Enterobacter cloacae*.

**Figure 8:**
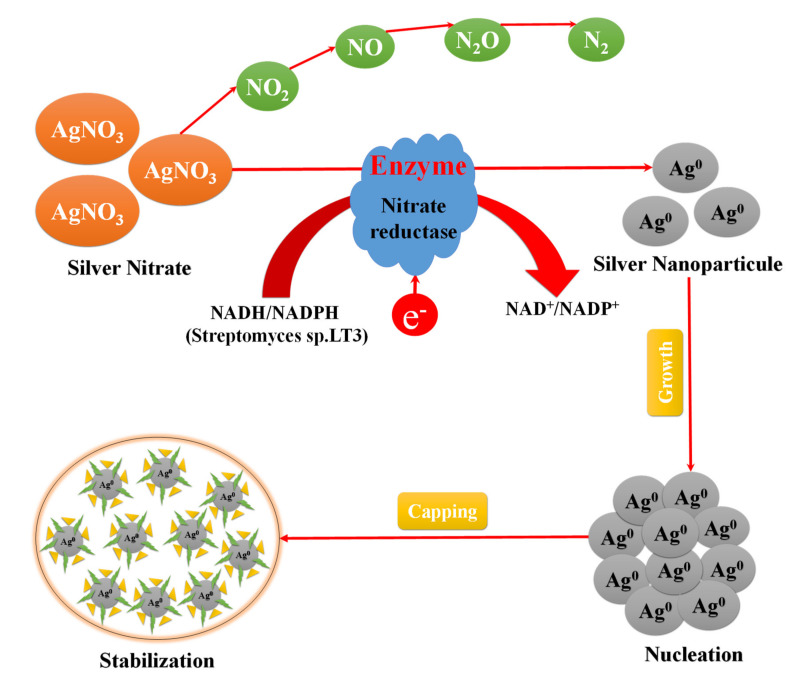
Proposed mechanism for Ag NPs synthesis by Streptomyces sp.LT3 involving NADH/NADPH dependent nitrate reductase enzyme, modified with permission from Ref. [106], Royal Society of Chemistry, 2019.

The first evidence of bacterial synthesis of Ag NPs was generated using a strain, the *Pseudomonas stutzeri* AG259 strain, meticulously isolated from silver mines [104]. A few microorganisms can endure metal particle concentrations and likewise grow under those conditions, and this wonder is because of their protection from that metal. The instruments engaged with the opposition are efflux frameworks, change of solvency and toxicity through reduction or oxidation, biosorption, bioaccumulation, extra-cell complex formation or precipitation of metals, and absence of explicit metal transport frameworks [105]. However, there is another perspective that these organisms can grow at lower concentrations, and their openness to higher concentrations of metal ions can incite toxicity. Figure 8 shows the proposed mechanism for silver nanoparticle synthesis by Streptomyces sp.LT3 involving an NADH/NADPH dependent nitrate reductase enzyme that converts Ag^+^ to Ag^0^ through electron shuttle enzymatic bioreducing agent.

#### 3.2.7. Green Synthesis Using Fungi

**Figure 9:**
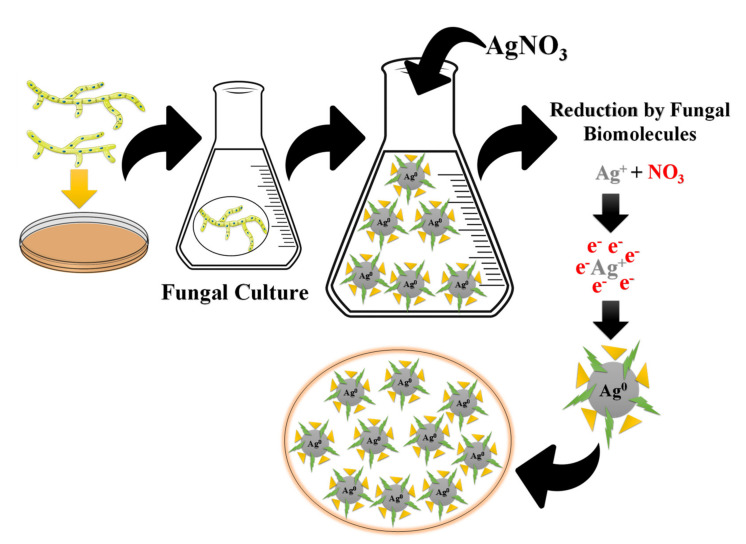
Mechanisms of biosynthesis of Ag NPs using fungi, modified with permission from Ref. [113], Frontiers, 2019.

Compared with bacteria, fungi can create more significant measures of NPs because they can emit more proteins, which directly means higher productivity of NPs [107]. The mechanism of silver nanoparticle creation by fungi follows the accompanying advances: catching Ag^+^ ions outside the fungal cells and reducing the silver ions by the enzymes present in the fungal framework [108] (as shown in Figure 9). The extracellular enzymes such as naphthoquinones and anthraquinones are said to work with the reduction. Regarding the case of *F. oxysporum*, it is accepted that the NADPH-dependent nitrate reductase and a van quinine extracellular cycle are answerable for nanoparticle formation [109]. Although the specific mechanism engaged with silver nanoparticle creation by fungi is not completely interpreted, it is accepted that the previously mentioned marvel is liable for the interaction. A significant downside of using microbes to synthesize Ag NPs is that it is an exceptionally sluggish cycle compared to plant extracts. Thus, the utilization of plant extracts to synthesize Ag NPs turns into a practical alternative.

In a recent study, Hietzschold et al. [110] showed that nanoparticle synthesis happened by the action of NADPH, with no requirement for the nitrate reductase protein. This is incredibly fascinating since it prompts various organisms to synthesize NPs without the vital condition of reductase compound creation. Nonetheless, Durán et al. [111] synthesized Ag NPs using *Fusarium oxysporum* and proposed that the reduction of silver ions was because of the action of the nitrate reductase protein and anthraquinones. Using sanitized nitrate reductase and phytochelatins from a similar fungus was tracked down that extracellular NADPH-dependent nitrate reductase enzymes and quinones were answerable for the formation of NPs [112].

### 3.3. Characterizations of Silver Nanoparticles

#### 3.3.1. Biological Characterizations

The beneficial properties of silver, limiting the spread of infection and improving daily hygiene, have been known and used for over 7000 years [51]. The compound was then continued to prevent water pollution or prevent eye infections in infants due to the application of AgNO_3_ [50]. Due to the release of Ag^+^ ions, its antibacterial properties have been recognized since bacteria were identified as the causative agents of infection. Many silver products were used until discovering antibiotics, including creams made from silver sulfadiazine and dressings made from silver leaves [114]. Silver is considered a “trace dynamic” element due to its antibacterial effect at low concentrations (range 0.1–10 mg L^−1^) [114]. According to Schierholz et al. [115], the minimum inhibitory concentration of most Gram-positive and Gram-negative bacteria is 0.5–10 ppm.

Bacteria in the presence of ionic silver can contain up to 100 Ag^+^ ions, even if they are highly diluted. This is the same number of digits as the number of enzymes present in the cell [51]. This phenomenon, called “in vivo accumulation,” explains the effectiveness of Ag^+^ ions at low concentrations [51]. Silver in metallic form is inert. However, when oxidized in contact with the atmosphere or humidity (Ag metal → Ag_2_O), the formed silver oxide dissolves, and Ag^+^ ions are released. These ions have a wide range of effects on Gram-positive and Gram-negative bacteria and yeast, fungi, and viruses. It is important to note that the killing effect of silver depends on the amount of Ag^+^ ions present in the medium and can interfere with microorganisms. Indeed, due to its high reactivity, silver can interact with proteins and salts in suspension media (e.g., AgCl formation, very sparingly soluble precipitates) and is an active amount for cells. Light also negatively impacts the biokilling efficiency of Ag^+^ ions by causing photoreduction of cations to metal atoms (Ag^0^) [116].

#### 3.3.2. Electronic Characterizations

In recent years, AgNP showed great attention in modern technological applications due to their high conductivity; conductive inks based on Ag NPs are widely utilized for electronic applications are based on Ag NPs [117,118]. As a noble metal, Ag NPs show a Localized Surface Plasmon Resonance (LSPR) as an optical phenomenon observed when the electromagnetic radiation excites the surface conducting electrons of MNPs, resulting in a coherent resonance oscillation of the particles. The location of the extinction maximum is highly dependent on the reflective index and dielectric properties of the surrounding environment and the adsorption of the molecules on the metal surface. This phenomenon can be utilized to detect the changes in the molecule-induced environment by measuring the visible and near-infrared wavelength regions [119]. Moreover, Ag NPs are used in solar cells to enhance light trapping and thus improve the assembled devices’ overall conversion efficiency. Ag NPs are employed in microelectronic materials due to significantly reduced melting points with increased surface energy. Ag NPs show a promising ability for microelectronic applications and can be applied as a conductive filler in electronically conductive adhesives [120]. The lower surface roughness of Ag NPs is an important feature to reduce the electrical losses at a higher frequency. Thus, the electrical conductors assembled with a thick film of silver nanoparticle reduce the electrical loss, giving better packing and fabricating antennas [121]. The electro reflectance (ER) effect of Ag NPs is one of the most promising features in optoelectronic and sensor applications. The change in the electronic charge stored on the particles alters the particle ensemble’s absorption spectrum. Typically, the ER effect for Ag NPs is 100 times stronger than for a bulk metal surface, which is readily discernible to the unaided eye [122].

## 4. Applications of Silver Nanoparticles

Over the last decades, the production of NPs has been increasing rapidly for applications in electronics, chemistry, biology, and almost all our daily life applications [123]. This is mainly due to their properties of being very small, close to the biomacromolecules and providing high surface area, rapid diffusion, and high reactivity in both liquid and gas phases [124,125]. Recently, Ag NPs have attracted attention in various applications such as biological, food, optoelectronics, electronic devices for energy conversion, electron field emission sources for emission displays, and surface-enhanced Raman properties [99,122,126,127]. Accordingly, utilizing Ag NPs in sensing, electronics, and photovoltaics, mainly for the excellent physio-chemical properties, will be reviewed in detail in the following sections.

### 4.1. Electronic Applications

Recently, great efforts have been dedicated to inkjet printing technology due to their wide applications in photovoltaics [128], radio-frequency identification devices [129], smart clothing [130], light-emitting diodes displays [131]. This interest is due to these advantages: (i) the inkjet technology could offer a digital and non-contact additive patterning that can be utilized for fabricating a wide range of materials, and (ii) low cost, which is the major factor for large scale production [132]. Hence, inkjet printing is proper for printing conductive tracks and patterns on various flexible substrates for assembling flexible electronics [133]. A lot of conductive materials, such as conductive polymers [134], carbon-based materials [135], and metal NPs [136], have been investigated as injecting materials and as a multi-directional printing of flexible and stretchable silver micro-electrodes [137]. However, the lower conductivity of both polymers and carbon (~10–102 S cm^−1^) comparable to that of metals (~104–105 S cm^−1^) and the high temperature (>250 °C) needed for printing are main limitations for flexible substrates [138]. Therefore, MNPs have significant attention as conductive inks [139,140]. The detailed procedures for printing nanomaterials have been reviewed in the literature [140,141]. Due to their high conductivity, conductive inks based on Ag NPs are widely utilized for electronic applications and are based on Ag NPs [117,118,137].

**Figure 10:**
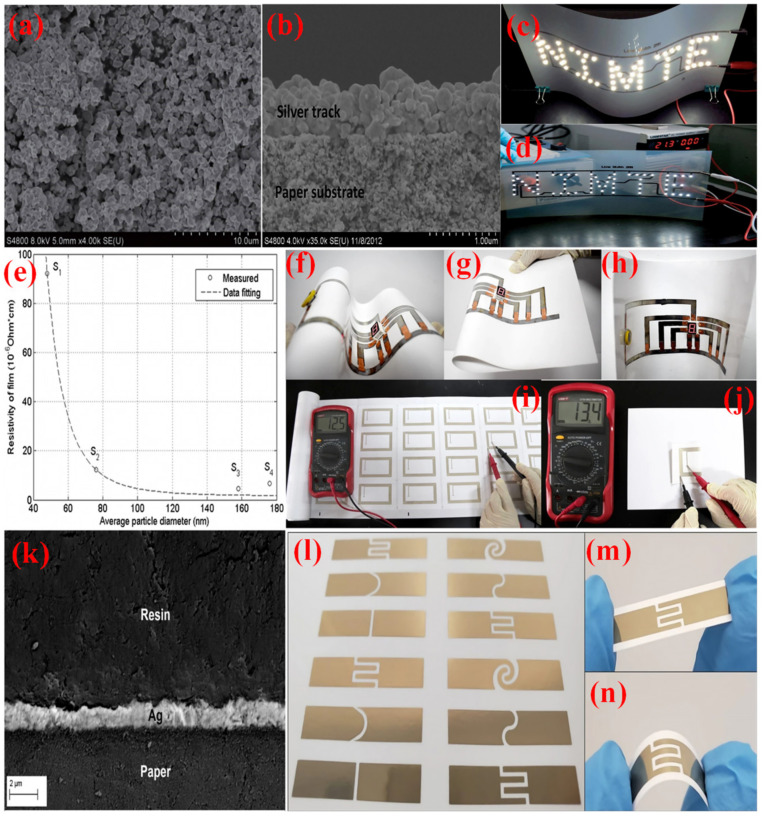
SEM images of Ag NPs powder (**a**), a cross-section of the printed silver track after heating at 50 °C (**b**), and LED device assembled by inkjet printing silver conductive circuits on photo paper (**c**) and PET (**d**) substrate, copied with permission from Ref. [118], Royal Society of Chemistry, 2014. The relationship between the resistivities of Ag NPs-based films and the average diameter of Ag NPs at 140 °C (**e**), hand-drawn 7 segment digital LED display circuit-bent in various shapes (**f**–**h**), and screen printed high-frequency RFID antenna before (**i**) and after folding (**j**), copied with permission from Ref. [117], Springer-Nature, 2019. SEM cross-section of ink Ag NPs on a photo paper and embedded in an epoxy resin (**k**), designs of the printed electrodes (**l**), with a closer view of design D-6 (**m**), and mechanical bending outwards (**n**) using the ink formulation, copied with permission from Ref. [142], Nature, 2020.

For instance, Shen et al. synthesized highly stable, homogeneous aqueous Ag NP inks by dispersing the Ag NPs in water [118]. These inks were printed on photo paper and polyethylene terephthalate (PET) substrates by a color printer. The printed patterns showed a significantly decreased resistivity to 3.7 µΩ cm when annealed at 180 °C, twice that of bulk silver. Furthermore, the printed patterns showed high conductivity (>20% of the bulk silver). A few years later, Mo et al. prepared Ag NPs with various radii (48–176 nm) as conductive ink for printed, electronic applications [117], as shown in Figure 10. It was found that the conductivity of the Ag NPs films significantly increased with increasing particle size. The prints on the art coated paper exhibited better flexibility compared to those on the photo paper. The RFID antenna was screen printed on art-coated paper using the Ag NPs-based conductive ink. The printed antenna with the conductive Ag line shows a resistance of 12.5 Ω after heating at 120 °C for 10 min, which is much lower than the commercially available screen-printed RFID antenna (~70 Ω). The printed RFID antenna also shows good resistance stability, changing from 12.5 to 13.4 Ω after face-to-face folding. Recently, Fernandes et al. [142] prepared seven conductive inks by Ag NPs, then printed them on a glossy photo paper (EPSON) substrate. As the temperature increases from 150 to 300 °C, the resistivity of the prepared inks reduced from 3.3 to 5.6 × 10^−6^ Ω cm, and the viscosity ranged from 3.7 to 7.4 mPa s, which is suitable for inkjet printing fabrication. Furthermore, the electrical impedance of all printed electrode pairs is less than 200 Ω, which is suitable for formulated inks for flexible electronic devices.

### 4.2. Sensing Applications

#### 4.2.1. Gas Sensing Applications

Developing fast, sensitive and selective gas sensors has received great attention in environmental monitoring, national security, and food safety applications [143]. Typically, the total performance of gas sensors strongly depends on the specific area surface of the materials utilized for detection gas. Thus nano-scale sensing is predictable to display improved sensing performance [39]. Typically, semi-conductive nano metal oxides such as ZnO and SnO_2_ exhibit high sensitivity and fast responses to some gases [144]. A relatively higher temperature (150–600 °C) required for maximum response is a big problem that restricts the practical applications in most areas [145]. Therefore, developing suitable nanomaterials to enhance the sensor response is highly recommended. Noble metal nanostructures are the most promising in this field due to LSPR. The LSPR is an optical phenomenon observed when the electromagnetic radiation excites the surface conducting electrons of MNPs, resulting in a coherent resonance oscillation of the particles. The location of the extinction maximum is highly dependent on the reflective index and dielectric properties of the surrounding environment and the adsorption of the molecules on the metal surface [119,146]. Therefore, the peak wavelength shift in the extinction maximum of NPs is used to fast detect molecule-induced changes surrounding the NPs. Thus, UV-Vis spectroscopy and the naked eye can observe the changes in the absorbance of the visible and near-infrared wavelength regions.

**Figure 11:**
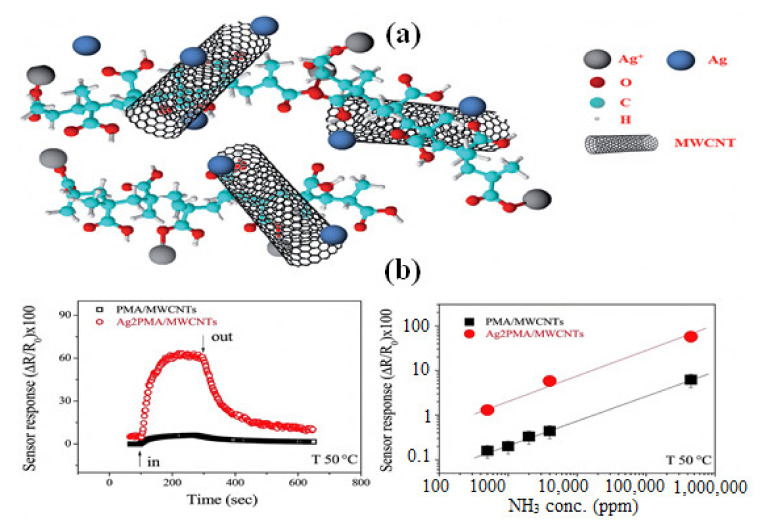
(**a**) Scheme for AgPMA/MWCNTs composite, (**b**) comparison between dynamic responses of PMA/MWCNTs and Ag2PMA/MWCNTs sensors to 44.7% of NH_3_ at 50 °C, copied with permission from Ref. [155], Royal Society of Chemistry, 2014.

There are many published articles on the use of LSPR sensing applications in the liquid phase to detect organic phosphorous pesticides [147] and ammonia [148]. Generally, SPR absorption of noble metal NPs is strong in the visible to near-infrared (IR) region. Moreover, SPR is particularly sensitive to its size, shape, composition, distance, and surroundings. Therefore, it displays a promising ability for various sensors. However, Ag NPs have higher extinction coefficients than gold nanoparticles (Au NPs) of the same size [149]. Due to this feature and high specific surface area, high catalytic, high crystallinity, and their electrical and optical properties, Ag NPs have been widely investigated as gas sensor applications [127,150]. This section will present the recent progress on Ag NPs as gas sensors for ammonia, methane, and hydrogen peroxide. Despite the wide use in industry, such as fertilizers, animal feed production, and manufacturing of paper and plastics, ammonia is a toxic material with a harmful effect on the human body as it can harm tissues and the immune system [151]. Ammonia is largely produced in deteriorating food and fruitbodies by various micro/macrofungi [152]. Thus, monitoring the concentration of ammonia in air and liquid in the atmosphere is extremely important. Recently, Ag NPs have been widely used to sense organic gases such as methane and ethanol [39,153,154]. Cannilla et al. [155] successfully prepared Ag NPs in a poly-methacrylic acid (PMA) matrix by a photo-induced reduction process followed by deposition on a ceramic substrate to sense ammonia gas in resistive base sensors. To improve the conductivity of the thin films to be suitable for sensing, the as-prepared Ag NPs/PMA were loaded with multi-walled carbon nanotubes (MWCNTs). The developed sensor shows the considerable ability to work at low temperature with a wide range of detection range and represent fast response/recovery times (Figure 11). Kumar et al. prepared face-centered cubic polyvinylpyrrolidone (PVP) capped Ag NPs at room temperature and a chemical reduction method [127]. The prepared sample showed an average size of ~22 nm, showing a conductive and metallic nature. As ammonium gas sensing, the synthesized NPs thin films showed the maximum sensitivity towards ammonia gas.

Ag NPs also show a promising activity as sensors for methane gas. Ghanbari et al. decorated Ag NPs with an average size of 29.3 nm on graphene via the chemical reduction method [150]. The sensing ability of the prepared Ag NPs/G was investigated against the methane gas in a resistive-based sensor. The results demonstrate that at methane concentrations less than 2000 mg L^−1^, the maximum response directly increases even at room temperature. Moreover, the Ag NPs/G shows a low limit of detection (LOD), highly selective, reversible, repeatable for methane sensing. Furthermore, Rithesh et al. prepared an Ag NPs/PVP/PVA composite with various concentrations of silver at room temperature [156]. The designed tapered plastic optical fiber gas sensor is utilized to mentor various concentrations of organic gases (ammonia, methanol, and ethanol) with a concentration ranging from 0 to 500 mg L^−1^. The Ag NPs/PVP/PVA showed better selectivity towards ammonia compared to the other gases.

**Figure 12:**
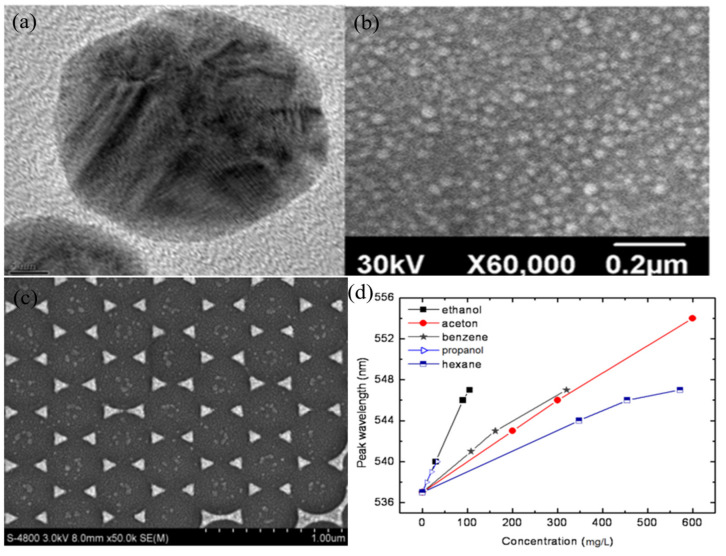
(**a**) TEM of Ag NPs/PVP/PVA composite, and (**b**) SEM of Ag NPs/PVP/PVA film on a glass plate, copied with permission from Ref. [156], Elsevier, 2015, (**c**) SEM of the triangular nanoprisms, and (**d**) calibration sensitivity curves of peak wavelength response to the concentrations of the tested vapors, copied with permission from Ref. [39], MDPI, 2011.

Moreover, among the investigated gases, the sensitivity of the proposed sensor against ammonia significantly increases with an increase in silver concentration. The sensor ability of Ag NPs against ethanol gas was investigated, as shown in Figure 12 [39]. Triangular Ag NPs were fabricated by a nanosphere lithography method; the obtained sample is arranged in an ideal hexangular array. The sensor of the prepared sample was investigated against vapors of ethanol, acetone, benzene, hexane, and propanol. However, ethanol exhibits the highest sensitivity (0.1 nm mg^−1^ L^−1^) with the lowest DOL (~10 mg L^−1^), as well as the ethanol vapor test process is also fast (~4 s) and reversible.

Banihashemian et al. successfully prepared single-wall carbon nanotubes (SWCNTs) decorated with Ag NPs using a reduction process [157]. The Ag NPs/SWCNTs reflect reasonable sensing for ethanol gas in the ambient temperature. The results revealed that the Ag NPs/SWCNTs sensor shows a response in the order of 7.1–45.3 at room temperature for 1–200 ppm (8–116 s response time and 7–38 s recovery time) LOD of 2 ppb with a full recovery response. Recently, Daniel et al. developed that adding Ag NPs to the hematite (α-Fe_2_O_3_) significantly improves the response of the assembled sensor against ethanol vapor at room temperature. This is mainly assigned to enhance the charge carrier density and the ethanol adsorption rate, thus increasing the selectivity and sensitivity. All these allowing quantifications of ethanol vapor in the 2–35 mg L^−1^ range with an excellent linear relationship.

#### 4.2.2. Hydrogen Peroxide Sensing Applications

In the last decade, hydrogen peroxide (H_2_O_2_) monitoring has gained importance due to its widely employed in many industrial, atomic power stations, medical sectors and its application as a disinfecting agent for water pools [158,159,160]. However, the high presence of H_2_O_2_ can cause various biological damages, leading to aging, neurodegeneration, and cancer [161,162]. Therefore, developing a high-response, cheap method is highly recommended for medical, pharmaceutical security, and environmental protection [161]. Various techniques, such as spectrophotometry [162,163], chemiluminescence [164], and electrochemistry [165], have been utilized to detect H_2_O_2_. These methods are categorized as enzymatic and non-enzymatic methods. In the case of enzymatic, the peroxidase is an illustrative enzyme for H_2_O_2_ exposure; however, both pH and temperature are limited.

**Figure 13:**
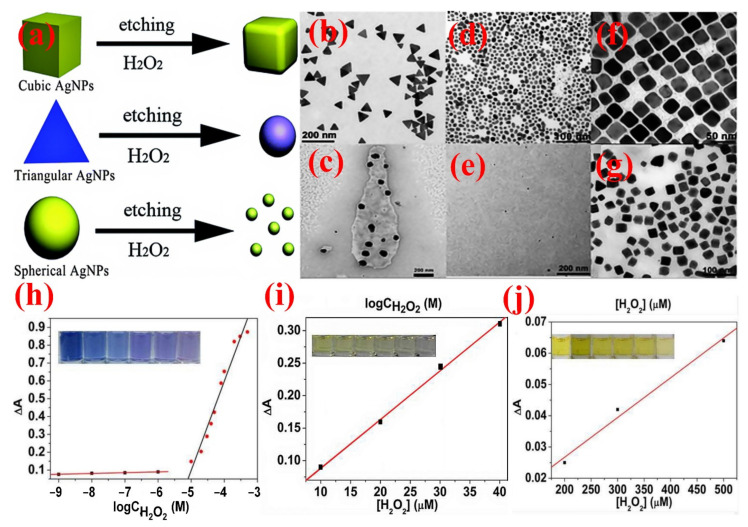
(**a**) Scheme for Ag NPs-H_2_O_2_ scheme for H_2_O_2_ sensing. TEM image of Ag NPs before etching, (**b**) triangular (**d**) spherical, and (**f**) cubic. TEM images of Ag NPs after etching, (**c**) triangular, (**e**) spherical, and (**g**) cubic. Plots of SPR peak change of (**h**) triangular, (**i**) spherical, and (**j**) cubic Ag NPs vs. concentrations of H_2_O_2_. (Inset the color change of Ag NPs in the presence of H_2_O_2_ with various concentrations), copied with permission from Ref. [167], Royal Society of Chemistry, 2016.

As mentioned above, noble metal NPs display strong SPR absorption with extreme sensitivity to the size, shape, composition, and surroundings suitable for colorimetric sensors in the range of visible to near-infrared region. For colorimetric assays, Ag NPs have a high ability toward the decay of H_2_O_2_ [166]. This reaction can mainly be sensed by colorimetric principles, as the colloidal Ag NPs exhibit a characteristic color from the LSPR. The alteration in both particle size and shape results from the incorporation of colloidal Ag NPs with H_2_O_2_ can be identified by determining the change in the absorption spectral at the wavelength of LSPR. For instance, Zhang et al. [167] investigated H_2_O_2_ via colorimetric detection using three different morphologies of Ag NPs (triangular, spherical, and cubic) as in Figure 13. The Ag NPs with various shapes reacted with H_2_O_2_, and the edges of Ag NPs had been etched. The change shape transformation induced visible color change, which was used for the quantitative determination of H_2_O_2_. The triangular Ag NPs display the highest sensitivity for a quite small level of H_2_O_2_ (5 mM), while the cubic Ag NPs display lower sensitivity. Recently, Srikhao et al. [168] prepared Ag NPs with an average particle size of 16.9 nm by using phenolic compound extracted from sugarcane leaves as a reducing agent. The results showed that 90 °C and stirring for 20 min are the optimum conditions for phenolic compound extraction. The prepared Ag NPs were evaluated as ammonia and H_2_O_2_ solution sensing by UV-Vis spectrophotometer and the naked eye. It observed that the Ag NPs sensitively detect both H_2_O_2_ and ammonia even at low concentrations.

Moreover, the prepared Ag NPs could detect these toxic agents even after two weeks. However, measuring in spectrophotometric methods requires some standard solutions and tools such as standardized cuvettes, microwell plates, and spectroscopic equipment. To overcome these limitations, Yoshikawa et al. utilized an optical technique for detecting H_2_O_2_ in their work Ag NPs deposited on a glass plate/Au NPs [166]. The Ag NPs chip diffracts incident light, and the diffraction efficiency is correlated with the amount of Ag NPs. By applying a drop of H_2_O_2_ onto the chip, the diffraction strength debilitates due to the decay of Ag NPs. A movable measurement technique of the diffraction intensity changes is assembled, and the H_2_O_2_ detection in a concentration range of 6.7–668 mmol L^−1^ in about 2 min by dropping the H_2_O_2_ solution onto the substrate.

The electrochemical technique is promising to detect H_2_O_2_ due to its simplicity, lower cost, ease of operation, and high sensitivity and selectivity compared to the other techniques [165]. Many electrochemical sensors based on metals and metal oxides-based complexes have been developed to overcome the high overpotential. Due to the high electron transfer rates, high catalytic activity toward reducing H_2_O_2_, and significantly decreasing overpotential at oxidizing and reducing agents, Ag NPs are widely utilized to fabricate these sensors. Zhan et al. [169] decorated Ag NPs on three-dimensional graphene (3DG) via the hydrothermal process as a sensing electrode for electrochemical detection of H_2_O_2_ in phosphate-buffered solutions. The electrochemical results approved that the Ag NPs-3DG based biosensor exhibits fast amperometric sensing, low LOD, wide linear responding range, and perfect selectivity for non-enzyme H_2_O_2_ detection. Recently, Maduraiveer et al. [170] utilized an electrochemical sensor for H_2_O_2_ by using Ag NPs introduced in a silicate matrix (APS(SG). The prepared APS(SG)-Ag NPs were deposited on glassy carbon (GC) electrode. The electron transfer behavior of the APS(SG)-Ag NPs was investigated by potassium ferricyanide ([FeCN)_6_]^3−^), methyl viologen (MV_2_) and ruthenium hexamine ([Ru (NH_3_)_6_]^3+^). The GC/APS(SG) electrode displayed a twofold increase in the peak currents and fast electron transfer kinetics toward [Fe (CN)_6_]^3−^ in comparison with the GC electrode. The GC electrode modified with APS(SG)-Ag NPs significantly improved electron transfer. The GC/APS(SG)-Ag NP electrode as an electrocatalytic sensor against H_2_O_2_ offered a reduction of H_2_O_2_ at less negative potential and showed the experimental low detection limit of 25.0 µM with the sensitivity of 0.042 µA/µM. Additionally, an APS(SG)-Ag NP-based sensor showed a fast response, good stability, and reproducibility.

### 4.3. Solar Cell Applications

Although the excellent research work in the conventional silicon and thin film-based solar cells, the production cost of solar cell electricity is still too high compared to the electricity supplied by fossil fuels. The efforts that have been made in recent decades can be categorized under two main strategies. One is to enhance the power conversion efficiency (PCE) and reduce the total production cost of silicon and thin film-based solar cells. At the same time, the other way involves fabricating alternative solar cells with low-cost production simultaneously with considerable total performance. The first one was achieved by using deposited thinner films or thinner silicon wafers. However, with the thinner films, the efficiency of light trapping will be reduced. To overcome this issue, introducing metallic NPs exhibited a promising way to enhance light trapping. This is mainly due to the excitation of surface plasmons. Ag NPs have a promising ability to increase absorption in wafer-based and thin-film silicon solar cells [171]. Bonsak et al. deposited a thin layer of Ag NPs as a trapping layer in silicon-based solar cells [172]. Ag NPs of various sizes were synthesized by the chemical reduction method using sodium borohydride and sodium citrate reducing agents. The photovoltaic study confirmed ~9% enhancement in the quantum efficiency at longer wavelengths. A few years later, Dzhafarov et al. reported the influence of Ag NPs on the optical and photovoltaic properties of silicon substrates, silicon solar cells, and glass [173]. Ag NPs layer was deposited by evaporation followed by thermal annealing. The deposition of Ag NPs on silicon substrates leads to 17.3% decreasing the reflectance at 600–1100 nm, at the same time resulting in 34% increases in the absorption in the wavelength ranger of 900–1100 nm. Moreover, Ag NPs deposited onto the front surface of the solar cells without antireflection coating increase the total efficiency by 39% comparing to the silicon cells without Ag NPs.

Another way that has also been taken into consideration is developing alternative solar cells with lower-cost materials. For instance, Gratzel group, in 1991, developed a kind of solar cell called dye-sensitized solar cell with a power conversion efficiency of about 7% [174]. However, the maximum power conversion is still lower than 15% [175,176,177,178]. The assembled device usually requires cost materials and a volatile liquid electrolyte unsuitable for the market in the current stage [175]. Replacing the expensive Pt with alternative materials with high catalytic activity is a promising way to reduce the total production cost of the assembled devices [179,180].

**Figure 14:**
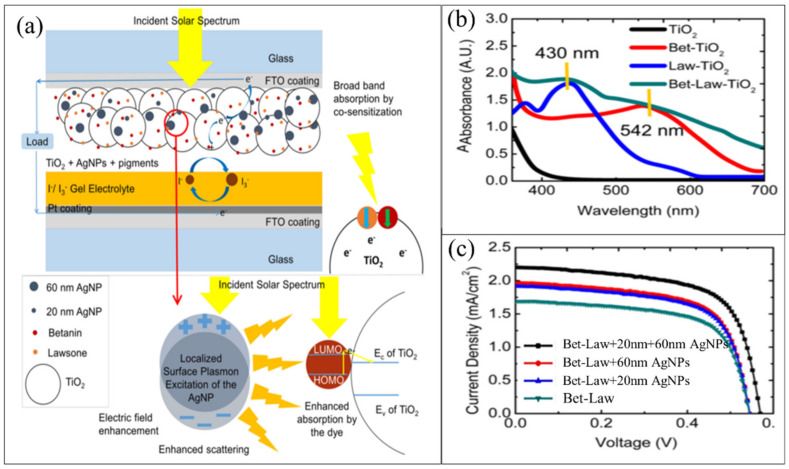
(**a**) Scheme of the betanin-lawsone sensitized solar cell with the bimodal distribution of 20 nm and 60 nm plasmonic Ag NPs incorporated in the photoanode. (**b**) absorption spectra of the sensitized TiO_2_ photoanodes, and (**c**) photocurrent density-voltage (J–V) curves of the assembled devices, copied with permission from Ref. [185], Nature, 2020.

Furthermore, utilizing polymers as an electrolyte is a promising technique to prevent the volatilization of electrolytes [181] and develop the photoanodes with various techniques. For example, Photiphitak et al. introduced Ag NPs to mesoporous titanium dioxide films using a photoreduction method [182]. TiO_2_/Ag NPs composite films were sensitized by N719 dye and inserted for fabricated dye-sensitized solar cells. The related device showed a PCE of 4.76%, higher than without adding Ag NPs (4.02%). Furthermore, Kislov et al. showed that Ag NPs on Gratzel cells enhance the total performance and show a noteworthy influence on the capacitive and transport properties of the assembled cells [183]. Recently, Saadmim et al. showed that the DSSCs assembled with photoanode supported with Ag NPs showed remarkable enhancing power conversion efficiency compared to that fabricated without Ag NPs [184]. Another work by Sreeja et al. achieved plasmonic enhancement in the two natural pigments (betanin-lawsone) co-sensitized solar cells by utilizing the bimodal size distribution of Ag NPs. The results significantly improve current density, voltage, and efficiency by 20.1%, 5.5%, and 28.6%, respectively. According to the Finite Difference Time Domain (FDTD) simulations, Ag NPs with a 20 and 60 nm diameter are best for enriched absorption by lawsone and betanin, respectively (see Figure 14). The FDTD simulations of the plasmonic photoelectrodes demonstrated 30% and 15% enhancement in the power absorption by betanin and lawsone at the LSPR peaks of the 60 and 20 nm Ag NPs, respectively. An average PCE of 1.02% was succeeded by the betanin-lawsone co-sensitized solar cell with the bimodal distribution of Ag NPs, compared to 0.793% achieved by the non-plasmonic solar cell. Moreover, electrochemical impedance spectroscopy approved that Ag NPs enriched the electron lifetime and diminished recombination rate, thus increasing the charge transfer.

## 5. Conclusions and Future Perspectives

This article presented the synthesis, properties, and applications of silver nanoparticles in detail. The recent progress on utilizing Ag NPs in electronics, sensing, and photovoltaics have been reviewed. Despite the great role of Ag NPs in sensing applications for detecting gases and vapors of some organic compounds and detecting hydrogen peroxide in industry and biosystems, the function of this effect needs more study to be fully understood. Furthermore, the long-term stability and sensitivity should be more developed by adjusting the preparation conditions, utilizing eco-friendly stabilizing agents. For solar cell application, Ag NPs can be introduced in both counter electrodes and an electrolyte to investigate the obtained total performance. Moreover, recycling Ag NPs from electronic wastes is an important issue from both environmental and economic points of view. This would help in developing Ag NP based nanomaterials more safe, biocompatible, and efficient for some vital applications in our life.

## Figures and Tables

**Table 1:** A list of various physically synthesized Ag NPs.

Physical Method	Applications	Shape	Size (nm)	Reference
Laser ablation	Antibacterial efficiency	Semi-spherical	14	[62]
		Spherical	13–32	[63]
	Antibacterial activity		5–30	[64]
	Catalytic degradation activity	Spherical-like	17	[65]
		Spherical	8–10	[66]
Thermal decomposition			40–50	[67]
			5–15	[68]
		Cubic/hexagonal	3.0–4.5	[69]
Small Ceramic Heater	Inhalation toxicity studies	Spherical	14	[70]

**Table 2:** Various plant extracts, bacteria, fungi, and biopolymers as bioreducing agents for the synthesis of Ag NPs.

Source	Bioreducing Agent of Silver Nitrate	The Mechanism for the Synthesis
Plants	Alkaloids, Terpenes, Steroids and Saponins, Flavonoids and Tannins, Alcohol, Phenolic Acids.	Electrostatic interaction between the functional groups of a respective constituent of plant extract and Ag^+^
Bacteria	*Bacillus Cereus, Bacillus licheniformis, Staphylococcus aureus, Enterobacteriaceae, Pantoeaananatis, Proteus* *mirabilis*	In the extra-cellular synthesis of Ag NPs by *Bacillus subtilis*, the synthesis of Ag NPs was observed in the reaction mixture after 6 h contact time at room temperature.
Fungi	Proteins, peptides enzymes, napthoquinones, NADH, NADPH, peptides, nitrogenous biomacromolecules,	Intracellular and extracellular synthesis of Ag NPs
Biopolymers	Cellulose, Chitosan, polypeptides, alginate, lignin, protein	Electrostatic interaction between Ag^+^ ion and polar groups attached to the polymer

**Table 3:** A list of synthesis Ag NPs using plant extracts with different sizes, shapes, characterization, and applications.

Plant Sources	Part	Applications	Operating Conditions	Size/Shape	Reference
Onion, tomato, *Acacia* *catechu*	Pieces	Photocatalytic application	16.987 g of AgNO_3_ in 1 L of distilled water, 10 mL extract, stirred for 10 min. and kept for 24 h at room temperature	32.1, 22.6, 14.5 nm, Spherical	[91]
*Curcuma longa* L.	Leaf	Antibacterial activity	10 mL of Curcuma longa L. leaf extrac, 90 mL of 1 mM AgNO_3_.	15–40 nm, spherical	[92]
*Phaseolus vulgaris* L.	Seeds	Photocatalytic activity, antimicrobial activity	Extract (1, 2, 3, and 4 mL), AgNO_3_ (0.01 M, 50 mL), (300 rpm) for 30 min	10–20 nm, spherical	[93]
*Solanum nigrum* L.	Leaves	Ecotoxicity Studies	1 mL leaf extract, AgNO_3_ (10^−3^ M, 50 mL), stirred continuously at room temperature	10–50 nm, spherical	[94]
f *Citrus* *reticulata*	Peels	Biocide and anticorrosion properties	1 mM AgNO_3_, ratio 1:1 with tangerine peels extract	39.6–56.1 nm, round	[95]
Apple	Fruits	Antibacterial activity	AgNO_3_ (1 mM, 90 mL), 10 mL of extract, at 70 °C.	45–110 nm, spherical shape	[96]
Clove	Buds	Antibacterial and antidiatom activity	AgNO_3_ (1 mM, 400 mL), 80 mL of extract, darkness for 24 h	9.42 nm	[97]
*Delonix regia*	Leaf	In vitro cytotoxicity and interaction studies with bovine serum albumin	Leaf extract and an aqueous solution of 1 mM AgNO_3_ (20:80, *V*/*V*).	72.77 nm, non-uniform	[98]

## Data Availability

Data is contained within the article.

## References

[1] Ayesha N., Khanna T., Vohora S. (1999). Silver preparations used in Indian systems of medicine: Neuropsychobehavioural effects. Indian J. Pharmacol..

[2] Alexander J.W. (2009). History of the Medical Use of Silver. Surg. Infect..

[3] Johnson N.A., Southerland M.R., Youngs W.J. (2017). Recent developments in the medicinal applications of silver-NHC complexes and imidazolium salts. Molecules.

[4] Fereshteh Jalilian A.C., Sadrjavadi K., Fattahi A., Shokoohinia Y. (2020). Green synthesized silver nanoparticle from Allium ampeloprasum aqueous extract: Characterization, antioxidant activities, antibacterial and cytotoxicity effects. Adv. Powder Technol..

[5] Nicole C., Mueller B.N. (2008). Exposure Modeling of Engineered Nanoparticles in the Environment. Environ. Sci. Technol..

[6] Chaloupka K., Malam Y., Seifalian A.M. (2010). Nanosilver as a new generation of nanoproduct in biomedical applications. Trends Biotechnol..

[7] Prabhu S., Poulose E.K. (2012). Silver nanoparticles: Mechanism of antimicrobial action, synthesis, medical applications, and toxicity effects. Int. Nano Lett..

[8] Heiligtag F.J., Niederberger M. (2013). The fascinating world of nanoparticle research. Mater. Today.

[9] Walter P., Welcomme E., Hallégot P., Zaluzec N.J., Deeb C., Castaing J., Veyssière P., Bréniaux R., Lévêque J.-L., Tsoucaris G. (2006). Early Use of PbS Nanotechnology for an Ancient Hair Dyeing Formula. Nano Lett..

[10] Johnson-McDaniel D., Barrett C.A., Sharafi A., Salguero T.T. (2013). Nanoscience of an Ancient Pigment. J. Am. Chem. Soc..

[11] Nabavifard S., Jalili S., Rahmati F., Vasseghian Y., Ali G.A.M., Agarwal S., Gupta V.K. (2020). Application of Dendrimer/Gold Nanoparticles in Cancer Therapy: A Review. J. Inorg. Organomet. Polym. Mater..

[12] Iravani S. (2011). Green synthesis of metal nanoparticles using plants. Green Chem..

[13] Aboelazm E.A.A., Ali G.A.M., Chong K.F. (2018). Cobalt oxide supercapacitor electrode recovered from spent lithium-ion battery. Chem. Adv. Mater..

[14] Fouad O.A., Makhlouf S.A., Ali G.A.M., El-Sayed A.Y. (2011). Cobalt/silica nanocomposite via thermal calcination-reduction of gel precursors. Mater. Chem. Phys..

[15] Ali G.A.M., Makhlouf A.S.H., Makhlouf A.S.H., Ali G.A.M. (2021). Fundamentals of Waste Recycling for Nanomaterial Manufacturing. Waste Recycling Technologies for Nanomaterials Manufacturing.

[16] Jeyaraj M., Gurunathan S., Qasim M., Kang M.-H., Kim J.-H. (2019). A Comprehensive Review on the Synthesis, Characterization, and Biomedical Application of Platinum Nanoparticles. Nanomaterials.

[17] Delphine Schaming H.R. (2015). Nanotechnology: From the ancient time to nowadays. Found Chem..

[18] Fouad O.A., Ali G.A.M., El-Erian M.A.I., Makhlouf S.A. (2012). Humidity sensing properties of cobalt oxide/silica nanocomposites prepared via sol-gel and related routes. Nano.

[19] Ali G.A.M., Fouad O.A., Makhlouf S.A., Yusoff M.M., Chong K.F. (2016). Optical and Electrochemical Properties of Co_3_O_4_/SiO_2_ Nanocomposite. Adv. Mater. Res..

[20] Ali G.A.M., Fouad O.A., Makhlouf S.A., Yusoff M.M., Chong K.F. (2014). Co_3_O_4_/SiO_2_ nanocomposites for supercapacitor application. J. Solid State Electrochem..

[21] Ali G.A.M., Fouad O.A., Makhlouf S.A. (2013). Structural, optical and electrical properties of sol-gel prepared mesoporous Co_3_O_4_/SiO_2_ nanocomposites. J. Alloys Compd..

[22] Danks A.E., Hall S.R., Schnepp Z. (2016). The evolution of ‘sol–gel’ chemistry as a technique for materials synthesis. Mater. Horiz..

[23] Livage J. (1997). Sol-gel processes. Curr. Opin. Solid State Mater. Sci..

[24] Abdel Ghafar H.H., Ali G.A.M., Fouad O.A., Makhlouf S.A. (2015). Enhancement of adsorption efficiency of methylene blue on Co_3_O_4/_SiO_2_ nanocomposite. Desalination Water Treat..

[25] Ethiraj A.S., Rhen D.S., Soldatov A.V., Ali G.A.M., Bakr Z.H. (2021). Efficient and recyclable Cu incorporated TiO2 nanoparticle catalyst for organic dye photodegradation. Int. J. Thin Film. Sci. Technol..

[26] Guglielmi M., Carturan G. (1988). Precursors for sol-gel preparations. J. Non-Cryst. Solids.

[27] Yao Y., Shu H., Tang B., Chen S., Lu Z., Xue W. (2015). Synthesis, Characterization and Application of Some Axially Chiral Binaphthyl Phosphoric Acids in Asymmetric Mannich Reaction. Chin. J. Chem..

[28] Thiagarajan S., Sanmugam A., Vikraman D. (2017). Facile methodology of sol-gel synthesis for metal oxide nanostructures. Recent Applications in Sol-Gel Synthesis.

[29] Thalji M.R., Ali G.A.M., Liu P., Zhong Y.L., Chong K.F. (2021). W_18_O_49_ nanowires-graphene nanocomposite for asymmetric supercapacitors employing AlCl_3_ aqueous electrolyte. Chem. Eng. J..

[30] Thalji M.R., Ali G.A.M., Algarni H., Chong K.F. (2019). Al^3+^ ion intercalation pseudocapacitance study of W_18_O_49_ nanostructure. J. Power Sources.

[31] Tao X.-Y., Ma J., Hou R.-L., Song X.-Z., Guo L., Zhou S.-X., Guo L.-T., Liu Z.-S., Fan H.-L., Zhu Y.-B. (2018). Template-Free Synthesis of Star-Like ZrO_2_ Nanostructures and Their Application in Photocatalysis. Adv. Mater. Sci. Eng..

[32] Bouafia A., Laouini S.E., Tedjani M.L., Ali G.A.M., Barhoum A. (2021). Green biosynthesis and physicochemical characterization of Fe_3_O_4_ nanoparticles using Punica granatum L. fruit peel extract for optoelectronic applications. Text. Res. J..

[33] Abderrhmane B., Salah Eddine L. (2021). Plant-Mediated Synthesis of Iron Oxide Nanoparticles and Evaluation of the Antimicrobial Activity: A Review. Mini-Rev. Org. Chem..

[34] Bouafia A., Laouini S.E., Ouahrani M.R. (2020). A review on green synthesis of CuO nanoparticles using plant extract and evaluation of antimicrobial activity. Asian J. Res. Chem..

[35] Bouafia A., Laouini S.E., Khelef A., Tedjani M.L., Guemari F. (2021). Effect of Ferric Chloride Concentration on the Type of Magnetite (Fe_3_O_4_) Nanoparticles Biosynthesized by Aqueous Leaves Extract of Artemisia and Assessment of Their Antioxidant Activities. J. Clust. Sci..

[36] Jung W.K., Kim S.H., Koo H.C., Shin S., Kim J.M., Park Y.K., Hwang S.Y., Yang H., Park Y.H. (2007). Antifungal activity of the silver ion against contaminated fabric. Mycoses.

[37] Arora S., Jain J., Rajwade M., Paknikar K.M. (2008). Cellular responses induced by silver nanoparticles: In vitro studies. Toxicol. Lett..

[38] Ma W., Yang H., Wang W., Gao P., Yao J. (2011). Ethanol vapor sensing properties of triangular silver nanostructures based on localized surface plasmon resonance. Sensors.

[39] Chen X., Schluesener H.J. (2008). Nanosilver: A nanoproduct in medical application. Toxicol. Lett..

[40] Farré M., Gajda-Schrantz K., Kantiani L., Barceló D. (2009). Ecotoxicity and analysis of nanomaterials in the aquatic environment. Anal. Bioanal. Chem..

[41] Scown T.M., Santos E.M., Johnston B.D., Gaiser B., Baalousha M., Mitov S., Lead J.R., Stone V., Fernandes T.F., Jepson M. (2010). Effects of Aqueous Exposure to Silver Nanoparticles of Different Sizes in Rainbow Trout. Toxicol. Sci..

[42] Scopus. https://www.scopus.com/.

[43] Lee P.C., Meisel D. (1982). Adsorption and Surface-Enhanced Raman of Dyes on Silver and Gold Sols. J. Phys. Chem..

[44] Van Hyning D.L., Zukoski C.F. (1998). Formation Mechanisms and Aggregation Behavior of Borohydride Reduced Silver Particles. Langmuir.

[45] Zielinska A., Skwarek E., Zaleska A., Gazda M., Hupka J. (2009). Preparation of silver nanoparticles with controlled particle size. Procedia Chem..

[46] Suzanne E., Howson A.R., Scott P., Bolhuis A., Brabec V., Clarkson G.J., Malina J. (2012). Optically pure, water-stable metallo-helical flexicate’ assemblies with antibiotic activity. Nat. Chem..

[47] Hsu S.L.-C., Wu R.-T. (2010). Preparation of Silver Nanoparticle with Different Particle Sizes for Low-Temperature Sintering. Int. Conf. Nanotechnol. Biosens. IPCBEE.

[48] Rodríguez-Sánchez L., Blanco M.C., López-Quintela M.A. (2000). Electrochemical Synthesis of Silver Nanoparticles. J. Phys. Chem. B.

[49] Khaydarov R.A., Khaydarov R.R., Gapurova O., Estrin Y., Scheper T. (2009). Electrochemical method for the synthesis of silver nanoparticles. J Nanoparticle Res.

[50] Dikovska A.O., Alexandrov M.T., Atanasova G.B., Tsankov N.T., Stefanov P.K. (2013). Silver nanoparticles produced by PLD in vacuum: Role of the laser wavelength used. Appl. Phys. A.

[51] Wei L., Lu J., Xu H., Patel A., Chen Z., Chen G. (2015). Silver nanoparticles: Synthesis, properties, and therapeutic applications. Drug Discov. Today.

[52] Burdușel A.-C., Gherasim O., Mogoantă L., Ficai A., Andronescu E., Grumezescu A.M. (2018). Biomedical Applications of Silver Nanoparticles: An Up-to-Date Overview. Nanomaterials.

[53] Chugh H., Sood D., Chandra I., Tomar V., Dhawan G., Chandra R. (2018). Role of gold and silver nanoparticles in cancer nano-medicine. Artif. Cells Nanomed. Biotechnol..

[54] Lee S.H., Jun B.H. (2019). Silver Nanoparticles: Synthesis and Application for Nanomedicine. Int. J. Mol. Sci..

[55] Tran Q.H., Nguyen V.Q., Le A.-T. (2013). Silver nanoparticles: Synthesis, properties, toxicology, applications and perspectives. Adv. Nat. Sci. Nanosci. Nanotechnol..

[56] Tsuji T., Iryo K., Ohta H., Nishimura Y. (2000). Preparation of Metal Colloids by a Laser Ablation Technique in Solution: Influence of Laser Wavelength on the Efficiencies of Colloid Formation. Jpn. J. Appl. Phys..

[57] Amendola V., Meneghettia M. (2009). Laser ablation synthesis in solution and size manipulation of noble metal nanoparticles. Phys. Chem. Chem. Phys..

[58] Martin H., Magnusson K.D., Malm J., Bovin J., Samuelson L. (1999). Gold nanoparticles: Production, reshaping, and thermal charging. J. Nanoparticle Res..

[59] Jung J.H., Oh H., Noh H.S., Ji J.H., Kim S.S. (2006). Metal nanoparticle generation using a small ceramic heater with a local heating area. Aerosol Sci..

[60] Tsuji T., Kakita T., Tsuji M. (2003). Preparation of nano-size particles of silver with femtosecond laser ablation in water. Appl. Surf. Sci..

[61] Menazea A.A. (2020). Femtosecond laser ablation-assisted synthesis of silver nanoparticles in organic and inorganic liquids medium and their antibacterial efficiency. Radiat. Phys. Chem..

[62] Elmira Solati M.M., Dorranian D. (2013). Effects of laser pulse wavelength and laser fluence on the characteristics of silver nanoparticle generated by laser ablation. Appl Phys. A.

[63] Menazeaa A.A., Ahmed M.K. (2020). Silver and copper oxide nanoparticles-decorated graphene oxide via pulsed laser ablation technique: Preparation, characterization, and photoactivated antibacterial activity. Nano-Struct. Nano-Objects.

[64] Ayman M., Mostafa A.A.M. (2020). Polyvinyl Alcohol/Silver nanoparticles film prepared via pulsed laser ablation: An eco-friendly nano-catalyst for 4-nitrophenol degradation. J. Mol. Struct..

[65] Wagener P., Ibrahimkutty S., Menzel A., Plech A., Barcikowski S. (2013). Dynamics of silver nanoparticle formation and agglomeration inside the cavitation bubble after pulsed laser ablation in liquid. Phys. Chem. Chem. Phys..

[66] Hosseinpour-Mashkani S.M., Ramezani M. (2014). Silver and silver oxide nanoparticles: Synthesis and characterizationby thermal decomposition. Mater. Lett..

[67] Goudarzi M., Mir N., Mousavi-Kamazani M., Bagheri S., Salavati-Niasari M. (2016). Biosynthesis and characterization of silver nanoparticles prepared from two novel natural precursors by facile thermal decomposition methods. Sci. Rep..

[68] Jeevanandam P., Srikanth C.K., Dixit S. (2010). Synthesis of monodisperse silver nanoparticles and their self-assembly through simple thermal decomposition approach. Mater. Chem. Phys..

[69] Ji J.H., Jung J.H., Yu J., Kim S.S. (2007). Long-Term Stability Characteristics of Metal Nanoparticle Generator Using Small Ceramic Heater for Inhalation Toxicity Studies. Inhal. Toxicol..

[70] Merga G., Wilson R., Lynn G., Milosavljevic B.H., Meisel D. (2007). Redox Catalysis on “Naked” Silver Nanoparticles. J. Phys. Chem. C.

[71] Oliveira M.M., Ugarte D., Zanchet D., Zarbin A.J.G. (2005). Influence of synthetic parameters on the size, structure, and stability of dodecanethiol-stabilized silver nanoparticles. J. Colloid Interface Sci..

[72] Bai J., Li Y., Du J., Wang S., Zheng J., Yang Q., Chen X. (2007). One-pot synthesis of polyacrylamide-gold nanocomposite. Mater. Chem. Phys..

[73] Pillai Z.S., Kamat P.V. (2004). What Factors Control the Size and Shape of Silver Nanoparticles in the Citrate Ion Reduction Method?. J. Phys. Chem. B.

[74] Bouafia A., Laouini S.E. (2020). Green synthesis of iron oxide nanoparticles by aqueous leaves extract of Mentha Pulegium, L.: Effect of ferric chloride concentration on the type of product. Mater. Lett..

[75] Gudimalla A., Jose J., Varghese R.J., Thomas S. (2021). Green Synthesis of Silver Nanoparticles Using Nymphae odorata Extract Incorporated Films and Antimicrobial Activity. J. Polym. Environ..

[76] Morales-Lozoya V., Espinoza-Gómez H., Flores-López Z.L., Sotelo-Barrera E.L., Núñez-Rivera A., Cadena-Nava R.D., Alonso-Nuñez G., Rivero I.A. (2021). Study of the effect of the different parts of Morinda citrifolia L. (noni) on the green synthesis of silver nanoparticles and their antibacterial activity. Appl. Surf. Sci..

[77] Kiani M., Rabiee N., Bagherzadeh M., Ghadiri A.M., Fatahi Y., Dinarvand R., Webster T.J. (2021). Improved Green Biosynthesis of Chitosan Decorated Ag- and Co_3_O_4_-Nanoparticles: A Relationship Between Surface Morphology, Photocatalytic and Biomedical Applications. Nanomed. Nanotechnol. Biol. Med..

[78] Vanlalveni C., Lallianrawna S., Biswas A., Selvaraj M., Changmai B., Rokhum S.L. (2021). Green synthesis of silver nanoparticles using plant extracts and their antimicrobial activities: A review of recent literature. RSC Adv..

[79] He Y., Li X., Zheng Y., Wang Z., Ma Z., Yang Q., Yao B., Zhao Y., Zhang H. (2018). A green approach for synthesizing silver nanoparticles, and their antibacterial and cytotoxic activities. New J. Chem..

[80] Laid T.M., Abdelhamid K., Eddine L.S., Bouafia A. (2021). Optimizing the biosynthesis parameters of iron oxide nanoparticles using central composite design. J. Mol. Struct..

[81] Abdullah J.A.A., Salah Eddine L., Bouafia A., Alonso-González M., Guerrero A., Romero A. (2020). Green synthesis and characterization of iron oxide nanoparticles by pheonix dactylifera leaf extract and evaluation of their antioxidant activity. Sustain. Chem. Pharm..

[82] Pirtarighat S., Ghannadnia M., Baghshahi S. (2019). Green synthesis of silver nanoparticles using the plant extract of Salvia spinosa grown in vitro and their antibacterial activity assessment. J. Nanostructure Chem..

[83] Gardea-Torresdey J.L., Gomez E., Peralta-Videa J.R., Parsons J.G., Troiani H., Jose-Yacaman M. (2003). Alfalfa Sprouts: A Natural Source for the Synthesis of Silver Nanoparticles. Langmuir.

[84] Laouini S.E., Bouafia A., Soldatov A.V., Algarni H., Tedjani M.L., Ali G.A.M., Barhoum A. (2021). Green Synthesized of Ag/Ag_2_O Nanoparticles Using Aqueous Leaves Extracts of Phoenix dactylifera L. and Their Azo Dye Photodegradation. Membranes.

[85] Krithiga N., Rajalakshmi A., Jayachitra A. (2015). Green Synthesis of Silver Nanoparticles Using Leaf Extracts of Clitoria ternatea and Solanum nigrum and Study of Its Antibacterial Effect against Common Nosocomial Pathogens. J. Nanosci..

[86] Yadav S., Khurana J.M. (2015). Cinnamomum tamala leaf extract-mediated green synthesis of Ag nanoparticles and their use in pyranopyrazles synthesis. Chin. J. Catal..

[87] Shankar S.S., Ahmad A., Sastry M. (2003). Geranium Leaf Assisted Biosynthesis of Silver Nanoparticles. Biotechnol. Prog..

[88] Mittal A.K., Chisti Y., Banerjee U.C. (2013). Synthesis of metallic nanoparticles using plant extracts. Biotechnol. Adv..

[89] Tarannum N., Gautam Y.K. (2019). Facile green synthesis and applications of silver nanoparticles: A state-of-the-art review. RSC Adv..

[90] Chand K., Cao D., Eldin Fouad D., Hussain Shah A., Qadeer Dayo A., Zhu K., Nazim Lakhan M., Mehdi G., Dong S. (2020). Green synthesis, characterization and photocatalytic application of silver nanoparticles synthesized by various plant extracts. Arab. J. Chem..

[91] Maghimaaa M., Alharbi S.A. (2020). Green synthesis of silver nanoparticles from Curcuma longa L. and coating on the cotton fabrics for antimicrobial applications and wound healing activity. J. Photochem. Photobiol. B Biol..

[92] Rani P., Kumar V., Singh P.P., Matharu A.S., Zhang W., Kim K.-H., Singh J., Rawat M. (2020). Highly stable AgNPs prepared via a novel green approach for catalytic and photocatalytic removal of biological and non-biological pollutants. Environ. Int..

[93] Jenifer A.A., Anjugam M., Malaikozhundan B., Iswarya A. (2020). Green Synthesis and Characterization of Silver Nanoparticles (AgNPs) Using Leaf Extract of Solanum nigrum and Assessment of Toxicity in Vertebrate and Invertebrate Aquatic Animals. J. Clust. Sci..

[94] Ituen E., Ekemini E., Yuanhua L., Singh A. (2020). Green synthesis of Citrus reticulata peels extract silver nanoparticles and characterization of structural, biocide and anticorrosion properties. J. Mol. Struct..

[95] Kazlagić A., Abud O.A., Ćibo M., Hamidović S., Borovac B., Omanović-Mikličanin E. (2020). Green synthesis of silver nanoparticles using apple extract and its antimicrobial properties. Health Technol..

[96] Lakhana M.N., Chena R., Shara A.H., Chanda K., Shahb A.H., Ahmeda M., Alic I., Ahmedd R., Liua J., Takahashia K. (2020). Eco-friendly green synthesis of clove buds extract functionalized silver nanoparticles and evaluation of antibacterial and antidiatom activity. J. Microbiol. Methods.

[97] Siddiquee M.A., Parray M.u.d., Mehdi S.H., Alzahrani K.A., Alshehri A.A., Malik M.A., Patel R. (2020). Green synthesis of silver nanoparticles from Delonix regia leaf extracts: In-vitro cytotoxicity and interaction studies with bovine serum albumin. Mater. Chem. Phys..

[98] Kanmani P., Lim S.T. (2013). Synthesis and structural characterization of silver nanoparticles using bacterial exopolysaccharide and its antimicrobial activity against food and multidrug resistant pathogens. Process Biochem..

[99] Klaus T., Joerger R., Olsson E., Granqvist C. (1999). Silver-based crystalline nanoparticles, microbially fabricated. Proc. Natl. Acad. Sci. USA.

[100] Priyadarshini S., Gopinath V., Priyadharsshini N.M., Ali D.M., Velusamy P. (2013). Synthesis of anisotropic silver nanoparticles using novel strain, Bacillus flexus and its biomedical application. Colloids Surf. B Biointerfaces.

[101] Saifuddin N., Wong W., Yasumira A.A.N. (2019). Rapid Biosynthesis of Silver Nanoparticles Using Culture Supernatant of Bacteria with Microwave Irradiation. E-J. Chem..

[102] Shahverdi A.R., Minaeian S., Shahverdi H.R., Jamalifar H., Nohi A. (2007). Rapid synthesis of silver nanoparticles using culture supernatants of Enterobacteria: A novel biological approach. Process Biochem..

[103] Haefeli C., Franklin C., Hardy K. (1984). Plasmid-determined silver resistance in Pseudomonas stutzeri isolated from a silver mine. J. Bacteriol..

[104] Husseiny M., El-Aziz M.A., Badr Y., Mahmoud M. (2007). Biosynthesis of gold nanoparticles using Pseudomonas aeruginosa. Spectrochim. Acta Part A.

[105] Gahlawat G., Choudhury A.R. (2019). A review on the biosynthesis of metal and metal salt nanoparticles by microbes. RSC Adv..

[106] Mohanpuria P., Rana N.K., Yadav S.K. (2008). Biosynthesis of nanoparticles: Technological concepts and future applications. J. Nanoparticle Res..

[107] Mukherjee P., Ahmad A., Mandal D., Senapati S., Sainkar S.R., Khan M.I., Parishcha R., Ajaykumar P.V., Alam M., Kumar R. (2001). Fungus-Mediated Synthesis of Silver Nanoparticles and Their Immobilization in the Mycelial Matrix: A Novel Biological Approach to Nanoparticle Synthesis. Nano Lett..

[108] Ahmad A., Mukherjee P., Senapati S., Mandal D., Khan M.I., Kumar R., Sastry M. (2003). Extracellular biosynthesis of silver nanoparticles using the fungus Fusarium oxysporum. Colloids Surf. B Biointerfaces.

[109] Hietzschold S., Walter A., Davis C., Taylor A.A., Sepunaru L. (2019). Does Nitrate Reductase Play a Role in Silver Nanoparticle Synthesis? Evidence for NADPH as the Sole Reducing Agent. ACS Sustain. Chem. Eng..

[110] Durán N., Marcato P.D., Durán M., Yadav A., Gade A., Rai M. (2011). Mechanistic aspects in the biogenic synthesis of extracellular metal nanoparticles by peptides, bacteria, fungi, and plants. Appl. Microbiol. Biotechnol..

[111] Kumar S.A., Abyaneh M.K., Gosavi S.W., Kulkarni S.K., Pasricha R., Ahmad A., Khan M.I. (2007). Nitrate reductase-mediated synthesis of silver nanoparticles from AgNO_3_. Biotechnol. Lett..

[112] Guilger-Casagrande M., Lima R.d. (2019). Synthesis of silver nanoparticles mediated by fungi: A review. Front. Bioeng. Biotechnol..

[113] Silver S., Phung L.T., Silver G. (2006). Silver as biocides in burn and wound dressings and bacterial resistance to silver compounds. J. Ind. Microbiol. Biotechnol..

[114] Schierholz J.M., Lucas L.J., Rump A., Pulverer G. (1998). Efficacy of silver-coated medical devices. J. Hosp. Infect..

[115] Kierans M., Staines A.M., Bennett H., Gadd G.M. (1991). Silver tolerance and accumulation in yeasts. Biol. Met..

[116] Mo L., Guo Z., Wang Z., Yang L., Fang Y., Xin Z., Li X., Chen Y., Cao M., Zhang Q. (2019). Nano-Silver Ink of High Conductivity and Low Sintering Temperature for Paper Electronics. Nanoscale Res. Lett..

[117] Shen W., Zhang X., Huang Q., Xu Q., Song W. (2014). Preparation of solid silver nanoparticles for inkjet printed flexible electronics with high conductivity. Nanoscale.

[118] Doria G., Conde J., Veigas B., Giestas L., Almeida C., Assuncao M., Rosa J., Baptista P.V. (2012). Noble metal nanoparticles for biosensing applications. Sensors.

[119] Umadevi M., Christy J. (2013). Optical, structural and morphological properties of silver nanoparticles and its influence on the photocatalytic activity of TiO2. Spectrochim. Acta A Mol. Biomol. Spectrosc..

[120] Chen D., Qiao X., Qiu X., Chen J. (2009). Synthesis and electrical properties of uniform silver nanoparticles for electronic applications. J. Mater. Sci..

[121] Alshehri A.H., Jakubowska M., Mlozniak A., Horaczek M., Rudka D., Free C., Carey J.D. (2012). Enhanced electrical conductivity of silver nanoparticles for high frequency electronic applications. ACS Appl. Mater. Interfaces.

[122] Khan I., Saeed K., Khan I. (2019). Nanoparticles: Properties, applications and toxicities. Arab. J. Chem..

[123] Kaur A., Gupta U. (2009). A review on applications of nanoparticles for the preconcentration of environmental pollutants. J. Mater. Chem..

[124] Anu Mary Ealia S., Saravanakumar M.P. (2017). A review on the classification, characterisation, synthesis of nanoparticles and their application. IOP Conf. Ser. Mater. Sci. Eng..

[125] Othman A.M., Elsayed M.A., Al-Balakocy N.G., Hassan M.M., Elshafei A.M. (2019). Biosynthesis and characterization of silver nanoparticles induced by fungal proteins and its application in different biological activities. J. Genet. Eng. Biotechnol..

[126] Kumar M., Devi P., Kumar A. (2017). Structural analysis of PVP capped silver nanoparticles synthesized at room temperature for optical, electrical and gas sensing properties. J. Mater. Sci. Mater. Electron..

[127] Liu T., Rong Y., Xiong Y., Mei A., Hu Y., Sheng Y., Jiang P., Hou X., Duan M., Guan Y. (2017). Spacer improvement for efficient and fully printable mesoscopic perovskite solar cells. RSC Adv..

[128] Kjellander B.K.C., Smaal W.T.T., Myny K., Genoe J., Dehaene W., Heremans P., Gelinck G.H. (2013). Optimized circuit design for flexible 8-bit RFID transponders with active layer of ink-jet printed small molecule semiconductors. Org. Electron..

[129] Park M., Im J., Shin M., Min Y., Park J., Cho H., Park S., Shim M.B., Jeon S., Chung D.Y. (2012). Highly stretchable electric circuits from a composite material of silver nanoparticles and elastomeric fibres. Nat. Nanotechnol..

[130] Haverinen H.M., Myllylä R.A., Jabbour G.E. (2009). Inkjet printing of light emitting quantum dots. Appl. Phys. Lett..

[131] Yin Z., Huang Y., Bu N., Wang X., Xiong Y. (2010). Inkjet printing for flexible electronics: Materials, processes and equipments. Chin. Sci. Bull..

[132] Singh M., Haverinen H.M., Dhagat P., Jabbour G.E. (2010). Inkjet printing-process and its applications. Adv. Mater..

[133] Xiong Z., Liu C. (2012). Optimization of inkjet printed PEDOT:PSS thin films through annealing processes. Org. Electron..

[134] Huang L., Huang Y., Liang J., Wan X., Chen Y. (2011). Graphene-based conducting inks for direct inkjet printing of flexible conductive patterns and their applications in electric circuits and chemical sensors. Nano Res..

[135] Mo L., Liu D., Li W., Li L., Wang L., Zhou X. (2011). Effects of dodecylamine and dodecanethiol on the conductive properties of nano-Ag films. Appl. Surf. Sci..

[136] Ahn B.Y., Duoss E.B., Motala M.J., Guo X., Park S.-I., Xiong Y., Yoon J., Nuzzo R.G., Rogers J.A., Lewis J.A. (2009). Omnidirectional Printing of Flexible, Stretchable, and Spanning Silver Microelectrodes. Science.

[137] Jung I., Jo Y.H., Kim I., Lee H.M. (2011). A Simple Process for Synthesis of Ag Nanoparticles and Sintering of Conductive Ink for Use in Printed Electronics. J. Electron. Mater..

[138] Kamyshny A., Magdassi S. (2014). Conductive Nanomaterials for Printed Electronics. Small.

[139] Huang Q., Zhu Y. (2019). Printing Conductive Nanomaterials for Flexible and Stretchable Electronics: A Review of Materials, Processes, and Applications. Adv. Mater. Technol..

[140] Nayak L., Mohanty S., Nayak S.K., Ramadoss A. (2019). A review on inkjet printing of nanoparticle inks for flexible electronics. J. Mater. Chem. C.

[141] Fernandes I.J., Aroche A.F., Schuck A., Lamberty P., Peter C.R., Hasenkamp W., Rocha T. (2020). Silver nanoparticle conductive inks: Synthesis, characterization, and fabrication of inkjet-printed flexible electrodes. Sci. Rep..

[142] Kohl D. (2001). Function and applications of gas sensors. J. Phys. D Appl. Phys..

[143] Kala P.V., Rao B.T., Srinivasarao K. (2019). Structural, optical and gas sensing properties of TiO2-MoO3 thin films. Int. J. Thin Film. Sci. Technol..

[144] Nikolic M.V., Milovanovic V., Vasiljevic Z.Z., Stamenkovic Z. (2020). Semiconductor Gas Sensors: Materials, Technology, Design, and Application. Sensors.

[145] Wan Q., Li Q.H., Chen Y.J., Wang T.H., He X.L., Li J.P., Lin C.L. (2004). Fabrication and ethanol sensing characteristics of ZnO nanowire gas sensors. Appl. Phys. Lett..

[146] Willets K.A., Van Duyne R.P. (2007). Localized surface plasmon resonance spectroscopy and sensing. Annu. Rev. Phys. Chem..

[147] Lin T.J., Huang K.T., Liu C.Y. (2006). Determination of organophosphorous pesticides by a novel biosensor based on localized surface plasmon resonance. Biosens. Bioelectron..

[148] Bewley R. (2019). FARO: A new type of neutron spectrometer with flux and resolution optimized. Rev. Sci. Instrum..

[149] Wei H., Chen C., Han B., Wang E. (2008). Enzyme Colorimetric Assay Using Unmodified Silver Nanoparticles. Anal. Chem..

[150] Ghanbari R., Safaiee R., Sheikhi M.H., Golshan M.M., Horastani Z.K. (2019). Graphene Decorated with Silver Nanoparticles as a Low-Temperature Methane Gas Sensor. ACS Appl. Mater. Interfaces.

[151] Behera S.N., Sharma M., Aneja V.P., Balasubramanian R. (2013). Ammonia in the atmosphere: A review on emission sources, atmospheric chemistry and deposition on terrestrial bodies. Environ. Sci Pollut. Res. Int..

[152] Imamura A., Yumoto T. (2008). Dynamics of fruit-body production and mycorrhiza formation of ectomycorrhizal ammonia fungi in warm temperate forests in Japan. Mycoscience.

[153] Rahman M.M., Khan S.B., Jamal A., Faisal M., Asiri A.M. (2012). Highly sensitive methanol chemical sensor based on undoped silver oxide nanoparticles prepared by a solution method. Microchim. Acta.

[154] Choudhury A. (2009). Polyaniline/silver nanocomposites: Dielectric properties and ethanol vapour sensitivity. Sens. Actuators B Chem..

[155] Cannilla C., Bonura G., Frusteri F., Spadaro D., Trocino S., Neri G. (2014). Development of an ammonia sensor based on silver nanoparticles in a poly-methacrylic acid matrix. J. Mater. Chem. C.

[156] Rithesh Raj D., Prasanth S., Vineeshkumar T.V., Sudarsanakumar C. (2015). Ammonia sensing properties of tapered plastic optical fiber coated with silver nanoparticles/PVP/PVA hybrid. Opt. Commun..

[157] Banihashemian S.M., Hajghassem H., Nikfarjam A., Azizi Jarmoshti J., Abdul Rahman S., Boon Tong G. (2019). Room temperature ethanol sensing by green synthesized silver nanoparticle decorated SWCNT. Mater. Res. Express.

[158] Martin B., Sedelmeier J., Bouisseau A., Fernandez-Rodriguez P., Haber J., Kleinbeck F., Kamptmann S., Susanne F., Hoehn P., Lanz M. (2017). Toolbox study for application of hydrogen peroxide as a versatile, safe and industrially-relevant green oxidant in continuous flow mode. Green Chem..

[159] Teong S.P., Li X., Zhang Y. (2019). Hydrogen peroxide as an oxidant in biomass-to-chemical processes of industrial interest. Green Chem..

[160] Kamrani M.S., Seifpanahi-Shabani K., Seyed-Hakimi A., Ali G.A.M., Agarwa S., Gupta V.K. (2019). Degradation of cyanide from gold processing effluent by H2O2, NaClO and Ca(ClO)2 combined with sequential catalytic process. Bulg. Chem. Commun..

[161] Ryoo D., Xu X., Li Y., Tang J.A., Zhang J., van Zijl P.C.M., Liu G. (2017). Detection and Quantification of Hydrogen Peroxide in Aqueous Solutions Using Chemical Exchange Saturation Transfer. Anal. Chem..

[162] Liu H., Ding Y., Yang B., Liu Z., Liu Q., Zhang X. (2018). Colorimetric and ultrasensitive detection of H_2_O_2_ based on Au/Co_3_O_4_-CeOx nanocomposites with enhanced peroxidase-like performance. Sens. Actuators B Chem..

[163] Teodoro K.B.R., Migliorini F.L., Christinelli W.A., Correa D.S. (2019). Detection of hydrogen peroxide (H_2_O_2_) using a colorimetric sensor based on cellulose nanowhiskers and silver nanoparticles. Carbohydr. Polym..

[164] Karimi A., Husain S.W., Hosseini M., Azar P.A., Ganjali M.R. (2018). Rapid and sensitive detection of hydrogen peroxide in milk by Enzyme-free electrochemiluminescence sensor based on a polypyrrole-cerium oxide nanocomposite. Sens. Actuators B Chem..

[165] Lee J.H., Huynh-Nguyen B.-C., Ko E., Kim J.H., Seong G.H. (2016). Fabrication of flexible, transparent silver nanowire electrodes for amperometric detection of hydrogen peroxide. Sens. Actuators B Chem..

[166] Yoshikawa H., Hieda K., Ikeda K., Tamiya E. (2019). Hydrogen peroxide detection with a silver nanoparticle grating chip fabricated by plasmonic plating. Anal. Methods.

[167] Zhang L., Li L. (2016). Colorimetric detection of hydrogen peroxide using silver nanoparticles with three different morphologies. Anal. Methods.

[168] Srikhao N., Kasemsiri P., Lorwanishpaisarn N., Okhawilai M. (2021). Green synthesis of silver nanoparticles using sugarcane leaves extract for colorimetric detection of ammonia and hydrogen peroxide. Res. Chem. Intermed..

[169] Zhan B., Liu C., Shi H., Li C., Wang L., Huang W., Dong X. (2014). A hydrogen peroxide electrochemical sensor based on silver nanoparticles decorated three-dimensional graphene. Appl. Phys. Lett..

[170] Maduraiveeran G., Kundu M., Sasidharan M. (2018). Electrochemical detection of hydrogen peroxide based on silver nanoparticles via amplified electron transfer process. J. Mater. Sci..

[171] Sangno R., Maity S., Mehta R.K. (2016). Plasmonic Effect Due to Silver Nanoparticles on Silicon Solar Cell. Procedia Comput. Sci..

[172] Bonsak J., Mayandi J., Thøgersen A., Stensrud Marstein E., Mahalingam U. (2011). Chemical synthesis of silver nanoparticles for solar cell applications. Phys. Status Solidi.

[173] Dzhafarov T.D., Pashaev A.M., Tagiev B.G., Aslanov S.S., Ragimov S.H., Aliev A.A. (2015). Influence of silver nanoparticles on the photovoltaic parameters of silicon solar cells. Adv. Nano Res..

[174] O’Regan B., Grätzel M. (1991). A low-cost, high-efficiency solar cell based on dye-sensitized colloidal TiO2 films. Nature.

[175] Gong J., Sumathy K., Qiao Q., Zhou Z. (2017). Review on dye-sensitized solar cells (DSSCs): Advanced techniques and research trends. Renew. Sustain. Energy Rev..

[176] Ahmed A.S.A., Xiang W., Gu A., Hu X., Saana I.A., Zhao X. (2018). Carbon black/silicon nitride nanocomposites as high-efficiency counter electrodes for dye-sensitized solar cells. New J. Chem..

[177] Ahmed A.S.A., Xiang W., Li Z., Amiinu I.S., Zhao X. (2018). Yolk-shell m-SiO_2_@ Nitrogen doped carbon derived zeolitic imidazolate framework high efficient counter electrode for dye-sensitized solar cells. Electrochim. Acta.

[178] Ahmed A.S.A., Xiang W., Amiinu I.S., Li Z., Yu R., Zhao X. (2019). ZnO-nitrogen doped carbon derived from a zeolitic imidazolate framework as an efficient counter electrode in dye-sensitized solar cells. Sustain. Energy Fuels.

[179] Ahmed A.S.A., Xiang W., Saana Amiinu I., Zhao X. (2018). Zeolitic-imidazolate-framework (ZIF-8)/PEDOT:PSS composite counter electrode for low cost and efficient dye-sensitized solar cells. New J. Chem..

[180] Ahmad S., Yum J.H., Butt H.J., Nazeeruddin M.K., Gratzel M. (2010). Efficient platinum-free counter electrodes for dye-sensitized solar cell applications. ChemPhysChem.

[181] Chen X., Tang Q., He B., Chen H. (2015). Graphene-incorporated quasi-solid-state dye-sensitized solar cells. RSC Adv..

[182] Photiphitak C., Rakkwamsuk P., Muthitamongkol P., Sae-Kung C., Thanachayanont C. (2011). Effect of Silver Nanoparticle Size on Efficiency Enhancement of Dye-Sensitized Solar Cells. Int. J. Photoenergy.

[183] Kislov D.A. (2015). Effect of Plasmonic Silver Nanoparticles on the Photovoltaic Properties of Graetzel Solar Cells. Phys. Procedia.

[184] Saadmim F., Forhad T., Sikder A., Ghann W., Ali M.M., Sitther V., Ahammad A.J.S., Subhan M.A., Uddin J. (2020). Enhancing the Performance of Dye Sensitized Solar Cells Using Silver Nanoparticles Modified Photoanode. Molecules.

[185] Sreeja S., Pesala B. (2020). Plasmonic enhancement of betanin-lawsone co-sensitized solar cells via tailored bimodal size distribution of silver nanoparticles. Sci. Rep..

